# Suppression of *Xo1*-Mediated Disease Resistance in Rice by a Truncated, Non-DNA-Binding TAL Effector of *Xanthomonas oryzae*

**DOI:** 10.3389/fpls.2016.01516

**Published:** 2016-10-13

**Authors:** Andrew C. Read, Fabio C. Rinaldi, Mathilde Hutin, Yong-Qiang He, Lindsay R. Triplett, Adam J. Bogdanove

**Affiliations:** ^1^Plant Pathology and Plant-Microbe Biology Section, School of Integrative Plant Science, Cornell UniversityIthaca, NY, USA; ^2^State Key Laboratory for Conservation and Utilization of Subtropical Agro-Bioresources, The Key Laboratory of Ministry of Education for Microbial and Plant Genetic Engineering, and College of Life Science and Technology, Guangxi UniversityNanning, China; ^3^Department of Plant Pathology and Ecology, The Connecticut Agricultural Experiment StationNew Haven, CT, USA

**Keywords:** transcription activator-like effector, plant disease resistance, defense suppression, *Xanthomonas oryzae*, bacterial blight of rice, bacterial leaf streak of rice, NLR protein, protein-protein interaction

## Abstract

Delivered into plant cells by type III secretion from pathogenic *Xanthomonas* species, TAL (transcription activator-like) effectors are nuclear-localized, DNA-binding proteins that directly activate specific host genes. Targets include genes important for disease, genes that confer resistance, and genes inconsequential to the host-pathogen interaction. TAL effector specificity is encoded by polymorphic repeats of 33–35 amino acids that interact one-to-one with nucleotides in the recognition site. Activity depends also on N-terminal sequences important for DNA binding and C-terminal nuclear localization signals (NLS) and an acidic activation domain (AD). Coding sequences missing much of the N- and C-terminal regions due to conserved, in-frame deletions are present and annotated as pseudogenes in sequenced strains of *Xanthomonas oryzae* pv. oryzicola (Xoc) and pv. oryzae (Xoo), which cause bacterial leaf streak and bacterial blight of rice, respectively. Here we provide evidence that these sequences encode proteins we call “truncTALEs,” for “truncated TAL effectors.” We show that truncTALE Tal2h of Xoc strain BLS256, and by correlation truncTALEs in other strains, specifically suppress resistance mediated by the *Xo1* locus recently described in the heirloom rice variety Carolina Gold. *Xo1*-mediated resistance is triggered by different TAL effectors from diverse *X. oryzae* strains, irrespective of their DNA binding specificity, and does not require the AD. This implies a direct protein-protein rather than protein-DNA interaction. Similarly, truncTALEs exhibit diverse predicted DNA recognition specificities. And, *in vitro*, Tal2h did not bind any of several potential recognition sites. Further, a single candidate NLS sequence in Tal2h was dispensable for resistance suppression. Many truncTALEs have one 28 aa repeat, a length not observed previously. Tested in an engineered TAL effector, this repeat required a single base pair deletion in the DNA, suggesting that it or a neighbor disengages. The presence of the 28 aa repeat, however, was not required for resistance suppression. TruncTALEs expand the paradigm for TAL effector-mediated effects on plants. We propose that Tal2h and other truncTALEs act as dominant negative ligands for an immune receptor encoded by the *Xo1* locus, likely a nucleotide binding, leucine-rich repeat protein. Understanding truncTALE function and distribution will inform strategies for disease control.

## Introduction

Diseases caused by members of the bacterial genus *Xanthomonas* constrain yield and reduce quality in a large variety of crop and ornamental plant species. In many cases, transcription activator-like (TAL) effector proteins of these bacteria play determinative roles in the host-pathogen interaction. Unique among the many types of effector proteins delivered into host cells through the type III secretion (T3S) pathway, TAL effectors localize to the nucleus and directly activate one or more genes by binding to effector-specific promoter sequences called effector binding elements (EBE; Doyle et al., 2013b). Targets include susceptibility (*S*) genes important for disease development, genes whose activation provides resistance, called executor *R* genes, and genes inconsequential to disease.

Distinguishing features of TAL effectors include an N-terminal T3S signal (Rossier et al., 1999; Szurek et al., 2002), a C-terminal activation domain (AD; Zhu et al., 1998), two functional nuclear localization signals (NLS; Yang and Gabriel, 1995; Van den Ackerveken et al., 1996; Zhu et al., 1998; Szurek et al., 2001), and their hallmark feature—a central region of polymorphic repeats (the “CRR”) that determines DNA binding specificity (Bonas et al., 1989; Boch et al., 2009; Moscou and Bogdanove, 2009; Deng et al., 2012; Mak et al., 2012). The repeats are typically 34 aa long, and individually form two-helix bundles that as a group assemble into a superhelix that wraps the DNA. The repeats each specify a single nucleotide in the EBE, contiguously, by virtue of a pair of neighboring amino acids at positions 12 and 13, called the repeat-variable diresidue (RVD). The RVD resides in the loop between the two helices of a repeat. The residue at position 12 stabilizes the loop and projects it into the major groove, where the 13th residue, called the “base-specifying residue” (de Lange et al., 2014), contacts the nucleotide. Common RVDs and their preferred nucleotides include HD:C, NG:T, NI:A, and NN:G/A. Some RVDs lack the second residue and are so designated using an asterisk, e.g., N^*^.

In the region immediately before the CRR, TAL effectors contain several positively charged residues (lysine and arginine) and four “cryptic” repeats (repeats −3 to 0) that structurally resemble the central repeats. These are posited to nucleate binding to DNA (Deng et al., 2012; Gao et al., 2012; Mak et al., 2012). Deletions of the cryptic repeats abolish binding (Miller et al., 2011; Meckler et al., 2013). TAL effectors exhibit a strong preference for T immediately 5′ of the EBE (Boch et al., 2009; Moscou and Bogdanove, 2009). The cryptic repeats, in particular a tryptophan residue (W232) in repeat −1, also play a yet incompletely understood role in that preference (Doyle et al., 2013a; Lamb et al., 2013; Schreiber and Bonas, 2014).

Some TAL effectors harbor within the CRR a repeat of unusual length. A few different lengths have been observed, ranging from 30 to 42 aa and appearing to have arisen from short internal duplications or deletions. Richter et al. (2014) found that a TAL effector containing a repeat of one of these lengths, which they refer to as “aberrant” repeats, will bind both its predicted EBE and a “frameshift” EBE missing a base pair at the corresponding position in the DNA. The structural basis for this apparent ability to disengage from the interaction is not known.

Overall, the code-like modularity of TAL effector-DNA recognition allows EBE prediction based on RVD sequence (Moscou and Bogdanove, 2009) and assembly of designer TAL effectors (dTALEs) with customized specificities for targeted gene activation (Boch et al., 2009). These capabilities have facilitated identification and characterization of both *S* and executor *R* genes in diseases of diverse crop plants (Moscou and Bogdanove, 2009; Strauss et al., 2012; Cernadas et al., 2014; Hu et al., 2014).

Bacterial leaf streak of rice and bacterial blight of rice are well-studied diseases in which TAL effectors play important roles. These diseases are caused by *Xanthomonas oryzae* pv. oryzicola (Xoc) and *X. oryzae* pv. oryzae (Xoo), respectively. Individual strains of Xoc and Xoo may deliver as many as two dozen or more different TAL effectors into rice cells during infection, though some (e.g., African Xoo strains) deliver fewer than 10. Bacterial leaf streak constrains rice yields in the tropics and subtropics of Asia, and increasingly elsewhere, including West Africa (Niño-Liu et al., 2006; Afolabi et al., 2014a,b; Poulin et al., 2014). Bacterial blight of rice is distributed similarly and in more temperate regions, and in many areas is the single most economically damaging disease of this staple crop (Niño-Liu et al., 2006).

Until recently (see below), no native major gene for resistance to bacterial leaf streak of rice had been identified, though the maize nucleotide-binding leucine-rich repeat (NLR) protein gene *Rxo1*, expressed as transgene in rice, is broadly effective since most Xoc strains express the avirulence effector AvrRxo1 recognized by Rxo1 (Zhao et al., 2004, 2005). In contrast, more than 40 *R* genes against bacterial blight of rice have been identified, and several have been cloned and characterized. These include: the TAL effector-induced executor genes *Xa10, Xa23, Xa27*, and likely *Xa7* (Yang et al., 2000; Gu et al., 2005; Tian et al., 2014; Wang et al., 2015); the pattern recognition receptor, receptor-like kinase genes *Xa21* and *Xa3*/*Xa26* (Song et al., 1995; Sun et al., 2004; Xiang et al., 2006); the *S* gene variants *xa13, xa25*, and *xa41*, which behave recessively as *R* genes (Yang et al., 2006; Liu et al., 2011; Hutin et al., 2015; Zhou et al., 2015); *xa5*, a TFIIA gamma subunit variant that impairs TAL effector-mediated gene activation (Iyer and McCouch, 2004; Gu et al., 2009; Huang et al., 2016); and *Xa1*, the only *R* gene for bacterial blight of rice so far shown to encode an NLR protein (Yoshimura et al., 1998).

In collaboration with others, we recently reported (Triplett et al., 2016) the identification and initial characterization of the *Xo1* resistance locus in a purified line of the heirloom rice variety Carolina Gold called Carolina Gold Select (Duitama et al., 2015). Not to be confused with *Xa1*, the *Xo1* locus is unique in that it is associated with resistance both to strains of Xoc and strains of Xoo. *Xo1*-mediated resistance is also unusual in that it is triggered by different TAL effectors from diverse *X. oryzae* strains. Surprisingly however, while Carolina Gold Select is resistant to each of several tested African strains of Xoc and Xoo, it was found susceptible to Asian strains tested. Recognition of TAL effectors by *Xo1* does not require the activation domain, a particular RVD sequence, or more than 3.5 repeats. In this way *Xo1* resembles the tomato bacterial spot resistance gene *Bs4*, which encodes an NLR protein (Schornack et al., 2004). *Xo1* was mapped to a 1.09 Mb interval containing ten NLR protein genes, of which eight are full-length genes encoding both the nucleotide-binding and the leucine-rich repeat regions (Triplett et al., 2016). We have since narrowed the interval to 1.01 Mb, leaving six full-length NLR protein genes among the candidate genes in the mapping interval (A. Huerta and LT, unpublished).

Here, we provide evidence that a new class of TAL effector, found in Asian strains of Xoc and Xoo, specifically suppresses *Xo1*-mediated resistance. Encoded by sequences previously dismissed as pseudogenes due to several in-frame deletions, these proteins have largely standard CRRs, but are missing part of the N-terminal region important for DNA binding, lack an AD, and exhibit only a single, candidate NLS sequence. Apparent orthologs in African strains and in a single Asian strain against which *Xo1* is effective harbor a disruptive IS element, a short CRR, and/or additional sequence polymorphisms. Because of the deletions, we refer to members of this new class of TAL effector as “truncTALEs,” for “truncated TAL effectors.” Many contain a 28 aa repeat, a length not reported previously. As a group truncTALEs exhibit a diversity of RVD sequences, indicating that they do not share a common predicted EBE. Indeed, we observed no DNA binding at all by a representative truncTALE in an *in vitro* assay. Our findings suggest a model in which suppression is mediated not by direct gene activation, but by another mechanism, possibly involving a dominant negative interaction with the product of *Xo1*. TruncTALEs explain the ability of Asian Xoo and Xoc strains to cause disease on Carolina Gold Select despite the effectiveness of *Xo1* against African strains. Given the strong likelihood that *Xo1* encodes an NLR protein, the prevalence in *X. oryzae* of members of this family of TAL effectors may also explain why so few NLR protein genes have been identified as bacterial leaf streak or bacterial blight resistance genes in rice.

## Materials and methods

### Plant material and growth conditions

Rice plants were grown in LC-1 soil mixture (Sungro, Bellevue, WA) in PGC15 growth chambers (Percival Scientific, Perry, IA) in trays ~60 cm below a combination of fluorescent and incandescent bulbs providing ~1000 μmoles/m^2^/s measured at 15 cm, under a cycle of 12 h light at 28°C and 12 h dark at 25°C.

### Bacterial strains and culture, plasmids, and primers

Bacterial strains and plasmids used are listed in Supplementary Table S1. Primers used are listed in Supplementary Table S2. *E. coli* strains were cultured in LB medium at 37°C. *X. oryzae* strains were grown in GYE (20 g/l glucose, 10 g/l yeast extract) at 28°C. Xoc BLS256 *tal2h* mutant strain M12 was created as part of a comprehensive TAL effector disruption library (Cernadas et al., 2014) by transformation with the suicide (non-replicative) plasmid pSM7 (Makino, 2005). PCR amplification and sequencing to map the M12 insertion were done as described (Cernadas et al., 2014), however, a new truncTALE specific reverse primer (B1826; Supplementary Table S2) was used to enable amplification from the 3′ end of a truncTALE gene. The crossover with the disrupted TAL effector gene in pSM7 (*tal7b* of Xoo strain PXO86) maps to the beginning of the coding sequence for the CRR of Tal2h. This may result in expression of a protein with the Tal2h N-terminus followed by four to five repeats of Tal7b.

### Construction of plasmids

#### Entry vectors

pAR008 allows assembly of a custom CRR coding sequence into a truncTALE (Tal2h) backbone coding sequence using our previously described Golden Gate kit for custom TAL effector construct assembly (Cermak et al., 2011). The vector was created in two steps with the NEBuilder HiFi DNA assembly kit (New England Biolabs, Ipswich, MA). First, Golden Gate entry vector pTAL1 (Cermak et al., 2011) was used as a template in a PCR reaction with primer pairs B1778/B1779 and B1781/B1782. The resulting intermediate vector was used as template in a second round of PCR with primer pair B2149/B2150, and Gibson assembled with coding sequence for the Tal2h N-terminal region amplified from genomic subclone pYH2 using primer pair B2147/B2148. Entry vector pAR009 is a version of pTAL1 that contains a silent mutation to abolish the *Sph*I site in the *tal1c* 3′ end, modified using Q5 mutagenesis (New England Biolabs) with primer pair B2171/B2172. Entry vector pAR012 is pAR008 further modified with a stop codon just prior to the coding sequence for the candidate NLS of Tal2h (RKRKSHD). It was generated in a Q5 mutagenesis reaction with primer pair B2168/B2169. An *Sph*I site and an *Aat*II site in each of these vectors can be used together to shuttle CRR coding sequences from existing TAL effector clones.

#### Chimeric TAL effector constructs

Coding sequences for Tal2h and Tal1c CRRs were transferred from pYH2 and pCS468, respectively, into the backbone coding sequences of pAR008, pAR009, and pAR012 using *Sph*I and *Aat*II. The complete coding sequences were then moved to *Xanthomonas* expression vector pKEB31 (Cermak et al., 2011) by Gateway LR reaction (ThermoFisher Scientific, Waltham, MA).

#### dTALE constructs

The Golden Gate kit (Cermak et al., 2011) was used to create Tal2h analog dTALE constructs dT1665, in which the 28 aa repeat at position 6 is replaced by a standard 34 aa repeat, and dT1666, which has the 28 aa repeat. Because Tal2h contains RVDs that are not represented in the kit, repeat modules were used in which the base-specifying residue matches the Tal2h sequence, and the last four RVDs of Tal2h were replaced arbitrarily with a single “HD” (Supplementary Table S3). For the 28 aa repeat a custom repeat module was synthesized by adapter cloning annealed oligonucleotides B1904 and B1905 into *Xba*I/*Xho*I-digested pNG7 (Cermak et al., 2011). Note that although pNG7 was used, once assembled, the amino acid changes are in repeat six. The dTALE constructs were assembled in pAR008 and moved to gateway destination vector pKEB31 for expression in *Xanthomonas*.

#### Protein expression constructs

Two protein expression constructs were initially created to facilitate the CRR swaps between Tal1c and Tal2h. For constructs containing the Tal1c N- and C-terminal coding regions, a fragment encoding truncated N- and C-termini (101 and 62 amino-acid residues from the ends, respectively), without the CRR, was amplified from pTAL1 using primer pair B2315/B2316 and inserted into vector pHis-parallel-SNAP using *Bam*HI and *Spe*I. Similarly, for protein expression constructs containing the Tal2h N- and C-terminal coding regions, a fragment encoding the complete N- and C-termini, without the repeat region, was amplified from pAR008 using primer pair B2359/B2360 and inserted into pHis-parallel-SNAP using *Bam*HI and *Spe*I. These backbone vectors were named pFR318 and pFR300, respectively. pHis-parallel-SNAP is a derivative of pHis-parallel1 (Sheffield et al., 1999). Constructs encoding full-length Tal2h (pFR303) or Tal2h with the Tal1c CRR (pFR320) were created by inserting the CRR coding sequences of Tal2h and Tal1c into pFR300 using *Nsi*I and *Aat*II. Constructs encoding Tal1c (pFR321) or Tal1c with the Tal2h CRR (pFR319) were created by inserting the CRR coding sequences of Tal1c and Tal2h into pFR318 in the same way. The final constructs each encode the protein fused to a 6xHistidine tag on the N-terminus. Note that proteins were not tagged with the SNAP protein since a stop codon was included in the primers used for PCR amplification.

### Leaf infiltration and virulence assays

Four to 6 weeks after planting, rice leaves were spot-infiltrated with bacterial suspensions in 10 mM MgCl_2_ at an approximate OD_600_ of 0.5 using a needleless syringe (Reimers and Leach, 1991). At least 15 spots were infiltrated on three plants per experiment, and experiments were carried out at least twice. Leaves were monitored and photographed at 48–72 h after infiltration for HR and at 10–12 days after infiltration for measuring lesion length. All infiltrations were repeated at least twice with similar results.

### Sequence alignments and feature annotation

Sequences of the N- and C-terminal regions of truncTALEs were aligned to those of Tal1c of Xoc strain BLS256 using T-Coffee (Notredame et al., 2000) accessed through the EMBL-EBI web portal (Goujon et al., 2010). Alignments are displayed using the “RTF new” output of BOXSHADE (http://www.ch.embnet.org/software/BOX_form.html). The candidate truncTALE NLS was identified using NLStradamus (Nguyen Ba et al., 2009).

### Protein expression and purification

Expression constructs were transformed into *E. coli* Rosetta pLysS (DE3) cells (EMD Millipore, Billerica, MA). Single colonies were grown overnight in 5 ml of LB supplemented with ampicillin and chloramphenicol. Cells were harvested by centrifugation, re-suspended in 5 ml of fresh media, and 0.5 ml used to inoculate 50 ml cultures. Cultures were incubated at 37°C, and protein expression was induced with 100 μM IPTG when OD_600_ reached ~0.8. Protein expression was carried out for 16 h at 15°C. Cells were harvested by centrifugation and the pellets were re-suspended in 5 ml of lysis buffer containing 20 mM Tris-HCl pH 8.0, 400 mM NaCl, and 5% glycerol and supplemented with protease inhibitors (ThermoFisher Scientific). Samples were sonicated on ice and lysate supernatants were added to 250 μl bed volume of TALON resin (Clontech Laboratories, Mountain View, CA) pre-equilibrated with lysis buffer. The resin was washed with 50 ml of lysis buffer, then 30 ml of high salt buffer (20 mM Tris-HCl pH 8.0, 2 M NaCl and 5% glycerol) to displace pre-bound DNA molecules (Meckler et al., 2013), and finally 20 ml of lysis buffer. The proteins were eluted with 5 ml of elution buffer containing 20 mM Tris-HCl pH 8.0, 400 mM NaCl, and 300 mM Imidazole. The eluates were concentrated down to 500 μL and dialyzed twice against 1 l of buffer containing 20 mM Tris-HCl pH 8.0, 400 mM KCl, 5 mM DTT, and 2 mM EDTA. Protein concentrations were calculated by SDS-PAGE using a BSA standard curve and different dilutions of each protein solution.

### Electrophoretic mobility shift assays

The EMSA protocol used was adapted from one published previously (Meckler et al., 2013). Probes used are listed in Supplementary Table S4. Probe oligonucleotides were purchased from Integrated DNA Technologies (Coralville, IA) as DNA duplexes with a biotin tag at the 5′ end of the complementary strand. The assays were performed using the Light Shift Chemiluminescent EMSA kit (ThermoFisher Scientific). Proteins were pre-incubated with 20 mM DTT at room temperature for 2 h to reduce disulfide bonds (Schreiber et al., 2015). Reactions were carried out with probes at 100 pM and proteins at 300 mM at room temperature for 50 min and mixed with sample buffer (10x sample buffer contains 25% ficoll and 2.5 mg/ml xylene cyanol in TE), and 10 μl of the total mixture was subjected to electrophoresis in a 1.3% agarose gel at 110 V for ~45 min at 4°C. The reaction mixtures were transferred to a nylon membrane pre-soaked in 0.5x TBE buffer at 100 V for 30 min at 4°C. The DNA was cross-linked to the nylon membrane immediately after transfer by placing the membrane face down in a Gel Doc imaging system (Bio-Rad, Hercules, CA) and exposing to UV for 12 min. DNA was detected using chemiluminescence following the kit protocol. Results were analyzed using Image Lab (BioRad).

## Results

### A family of TAL effectors in *X. oryzae* with conserved N- and C-terminal deletions

**Figure 1:**
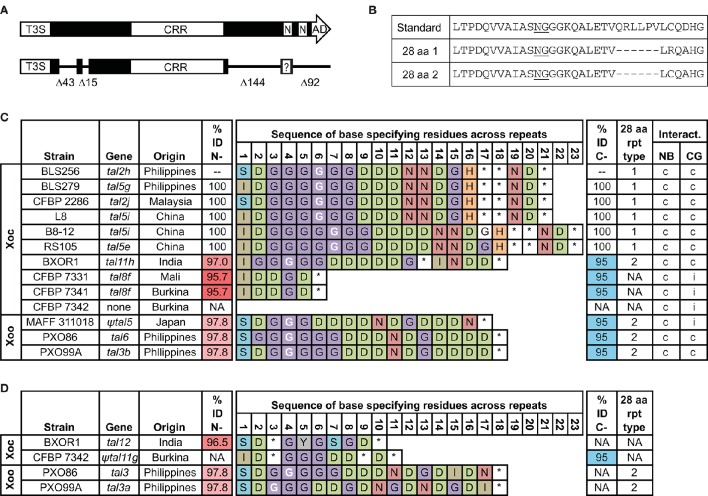
**Features and distribution of truncTALE and truncTALE-related sequences across *X. oryzae* strains in relation to the ability to suppress *Xo1*-mediated resistance. (A)** Schematic of a standard TAL effector (top) and a truncTALE showing the conserved N-and C-terminal deletions labeled by the number of amino acids missing. In both TAL effectors and truncTALEs, the length of the CRR varies; for this reason and to conserve space, the CRR is not drawn to scale. T3S, type III secretion signal; CRR, central repeat region; N, NLS; AD, activation domain. In truncTALEs, amino acid substitutions in the first NLS create a new, candidate NLS sequence (question mark), shifted N-terminally one amino acid (see Supplementary Figure S1). **(B)** Amino acid (aa) sequences of a standard TAL effector repeat and two types of 28 aa repeats found in truncTALEs. **(C)** TruncTALEs across *X. oryzae* pv. oryzicola (Xoc) and *X. oryzae* pv. oryzae (Xoo) strains and compatibility of each strain with rice varieties Nipponbare (NB), which lacks *Xo1*, and Carolina Gold Select (CG), which carries it. Percent identity (% ID) is to Tal2h, and like-colored cells indicate an identical aa sequence. A base-specifying residue (the second residue of the RVD; asterisk, missing amino acid) in white font indicates a 28 aa repeat at that position. NA, not applicable. A compatible (c) interaction is one that leads to disease; an incompatible (i) interaction results in HR (see text). The truncTALE gene in Xoo strain MAFF 311018, *tal5*, contains an insertion sequence element in the promoter region that almost certainly prevents expression and renders it a pseudogene, denoted by the prefix “ψ.” **(D)** Other, truncTALE-related sequences in some Xoc and Xoo strains. The *tal12, tal3*, and *tal3a* genes, respectively of Xoc strain BXOR1 and Xoo strains PXO86 and PXO99A, each encode a protein with a Tal2h-like N-terminus and a standard TAL effector C-terminus except for a few substitutions in the first NLS and a premature stop just after that. In Xoc strain CFBP 7342, ψtal11g is a clearly pseudogenized version of the apparent inverse, a recombinant with the 5′ end of a standard TAL effector gene and the 3′ end of a truncTALE gene, in which a frameshift mutation occurs at bp 88 and an insertion sequence element at bp 152. An open reading frame exists downstream of the insertion sequence element, but it is likely not expressed, and, with its putative start corresponding to amino acid position 75 of a standard TAL effector, it would lack any T3S. TruncTALE and truncTALE-related sequences were extracted from the respective complete genome sequences (Ochiai et al., 2005; Salzberg et al., 2008; Booher et al., 2015). Genome coordinates are provided in Supplementary Table S5.

We published the complete genome sequence of Philippines Xoc strain BLS256 in 2011 (Bogdanove et al., 2011). In it we annotated 26 TAL effector genes and two TAL effector pseudogenes. One of the pseudogenes, *tal2h'*, actually comprises an intact ORF. The ORF encodes a TAL effector variant that we presumed to be non-functional: though it retains its T3S signal and a CRR, the variant suffers two moderate-size deletions in the N-terminal region that remove part of the cryptic repeats and several of the positive residues posited to nucleate DNA binding, and two large deletions in its C-terminal region (Figure 1A) that remove the second NLS and the AD. Further, amino acid substitutions at the first NLS ablate it, although they do create a new, candidate NLS sequence shifted 1 aa toward the N-terminus (Supplementary Figure S1). The variant is also distinguished by the presence of an aberrant repeat of a novel length, 28 aa, at position 6 in the CRR (Figures 1B,C). More recently, we assembled whole genome sequences for nine more Xoc strains from diverse locations (Booher et al., 2015; Wilkins et al., 2015). All but one, the African strain CFBP 7342, encode similar variants, with the same N- and C-terminal deletions. A protein of this type is also encoded in each of several Xoo genomes we examined, though in one, MAFF 311018, it is likely not expressed (see below; Figure 1C). Because of the conserved overall structure, we surmised that *tal2h'* is in fact not a pseudogene. We designate its product as Tal2h, and based on their shortened N- and C-terminal regions, we refer to the family of proteins in *X. oryzae* that share the deletions found in Tal2h as truncated TAL effectors, or “truncTALEs.” Respective genome coordinates for each of the truncTALE coding sequences are provided in Supplementary Table S5. We have not found members of this family encoded in the published genomes of *Xanthomonas* species other than *X. oryzae*.

The CRRs across all truncTALEs range in length from six RVDs (in the two African Xoc strains examined that harbor a truncTALE) to 23 RVDs (in two Chinese Xoc strains). Tal2h has 21 RVDs. Based on the length variation and the sequences of base specifying residues of each protein, the CRRs appear to have diverged from an ancestral repeat array by repeat duplications and excisions, resulting in a diversity of predicted DNA targeting specificities (Figure 1C). Except for the truncTALEs in the two African Xoc strains, which comprise only standard 34 aa repeats, each truncTALE harbors a single 28 aa repeat, at position 4, 6, or 7. Every 28 aa repeat carries the RVD “NG,” or in the case of the truncTALE in the Indian Xoc strain examined, “HG.”

A single aa polymorphism in the backbone of the 28 aa repeat (R24C) distinguishes the East Asian Xoc truncTALEs from the Indian Xoc truncTALE and the Xoo truncTALEs (Figures 1B,C). A small number of polymorphic residues in the C-terminal region similarly distinguish the East Asian Xoc truncTALEs from those of the Indian and the African Xoc strains and the Xoo strains, and amino acid polymorphisms in the N-terminal region further distinguish those latter groups from one another (Figure 1C and Supplementary Figure S1). These relationships are consistent with the hypothesis based on the CRRs that the proteins diverged from a common ancestral truncTALE and diverged with the pathovars and pathovar subgroups. The Indian Xoc truncTALE being more similar to the Xoo truncTALEs is a possible exception and may represent an example of horizontal transfer.

### Other, TruncTALE-related sequences in Xoc and Xoo strains

The absence of a truncTALE from the African Xoc strain CFBP 7342 appears to have resulted, in part, from recombination. Pseudogene ψtal11g of this strain (Figure 1D) exhibits a partial open reading frame downstream of a frameshift mutation at bp 88 and an insertion sequence element at bp 152 in the 5′ end of a standard TAL effector gene, followed in-frame by CRR and C-terminal region coding sequences typical of a truncTALE gene (Booher et al., 2015). In Xoo strain MAFF 311018, the truncTALE coding sequence, which is otherwise very similar to those of Xoo strains PXO86 and PXO99A (Figure 1C), is preceded immediately by an IS element that almost certainly disrupts expression and renders it also a pseudogene, ψtal5.

In the genomes of the Indian strain BXOR1 and in two Xoo strains, PXO86 and PXO99A, a gene encoding a TAL effector variant with a truncTALE N-terminal region and a distinct C-terminal region is also present (Figure 1D and Supplementary Figure S1). The C-terminal region is that of a standard TAL effector except that it terminates due to a premature stop codon immediately after the coding sequence for the first NLS. Downstream of the stop codon the sequence is intact, corresponding to that of a standard TAL effector C-terminal coding sequence (Supplementary Figure S1). As in truncTALEs, substitutions in the NLS reconstitute a new, candidate one (ASRRKRS). The evolutionary origin of these genes is unclear, but each is associated with an apparent local or regional duplication in the genome (Booher et al., 2015; Wilkins et al., 2015), and each appears to be recombinant (Booher et al., 2015). In the Xoo strains, the predicted CRRs harbor a 28 aa repeat, but overall similarity of the RVDs to the truncTALEs in these strains breaks down early in the CRR. In the Indian Xoc strain, there is no 28 aa repeat, and the RVD sequence of the CRR is dissimilar throughout relative to the truncTALE in that strain (Figure 1D).

### Specific suppression of *Xo1*-mediated resistance by TruncTALE Tal2H

**Figure 2:**
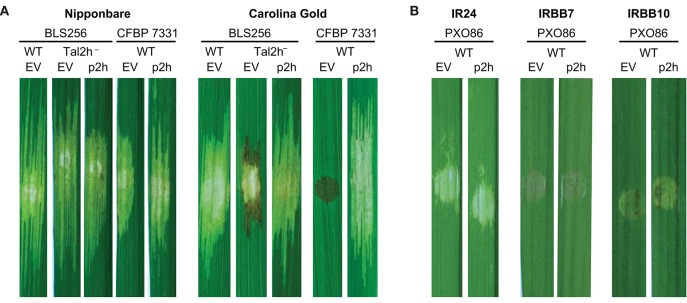
**Specific suppression of *Xo1*-mediated resistance by truncTALE Tal2h. (A)** Tal2h suppresses resistance mediated by *Xo1*. Leaves of rice cultivar Nipponbare, which lacks *Xo1*, or cultivar Carolina Gold Select, which carries it, were photographed with trans-illumination (on a light box) 10 days after inoculation by syringe infiltration with suspensions of Xoc BLS256 *tal2h* mutant M12 (*tal2h*^−^) or Xoc CFBP 7331 wild type (WT), each transformed either with an empty plasmid vector (EV) or with that vector carrying *tal2h* (p2h). BLS256 wild type carrying the empty vector was included for reference. The lesion on Carolina Gold Select caused by the *tal2h* mutant of BLS256 carrying the empty vector expanded for the first few days then developed HR-like necrosis, dark at the margins, and ceased expanding. The necrosis on Carolina Gold Select caused by CFBP 7331 carrying the empty vector developed within 48 h and was limited to the site of inoculation. **(B)** Tal2h does not suppress resistance mediated by *Xa7* or *Xa10*. Xoo strain PXO86, which triggers both *Xa7* and *Xa10*-mediated resistance, was transformed with the empty vector or p2h and inoculated to rice cultivars IRBB7, which contains *Xa7*, and IRBB10, which contains *Xa10*. Inoculations to cultivar IR24, which contains neither resistance gene, were included for reference. Leaves were photographed on a light box at 5 days after inoculation.

To probe the possible function of truncTALEs, we identified a *tal2h* marker exchange mutant of Xoc BLS256 from a previously generated library (Cernadas et al., 2014) and assayed it for any difference from wild type in compatibility (qualitative ability to cause disease) or virulence (quantitative ability to cause disease) when inoculated to rice leaves by spot infiltration (Reimers and Leach, 1991; Figure 2A). We tested the fully sequenced cultivar Nipponbare and the heirloom variety Carolina Gold Select, in both of which wildtype BLS256 is fully virulent. We chose Carolina Gold Select hypothesizing that the differences in truncTALEs between the Asian and African Xoc strains, and the absence of a truncTALE from the African strain CFBP 7342, might relate to the contrasting compatibility of Asian strains with this variety and incompatibility of African strains with it due the presence of the *Xo1* resistance gene (Triplett et al., 2016). In Nipponbare leaves, the *tal2h* mutant strain caused expanding lesions indistinguishable from those caused by the wildtype strain, but in Carolina Gold Select leaves, within 5 days of inoculation, it elicited necrosis typical of the resistance-associated hypersensitive reaction (HR) at the lesion margin that limited further lesion expansion. The *tal2h* gene on a plasmid fully complemented the mutation, eliminating the HR-like necrosis and restoring full-length lesions in Carolina Gold Select.

We also assayed the effect of heterologous expression of *tal2h* in the African strain CFBP 7331. CFBP 7331 carrying the empty plasmid vector, as expected, was fully virulent in Nipponbare and triggered *Xo1*-mediated resistance in Carolina Gold Select, with dark necrosis characteristic of the HR, limited to the spot of inoculation (Triplett et al., 2016); expressing Tal2h however, CFBP 7331, while unchanged in Nipponbare, became fully compatible with Carolina Gold Select, causing lesions similar in length to those on Nipponbare rather than the HR (Figure 2A). Thus, Tal2h suppresses *Xo1*-mediated resistance. We hypothesize that the initial lesion expansion resulting from inoculation of the BLS256 *tal2h* mutant results from residual suppression due to expression of a partial Tal2h protein from the marker-exchanged locus (see Section Materials and Methods) or the presence of a distinct, weak suppressor of resistance.

We next inoculated each of the Xoc and Xoo strains examined to Carolina Gold Select leaves (Figure 1C). Each of the strains was compatible, causing disease, except the three African Xoc strains and Xoo strain MAFF 311018. As presented above, one of the African strains lacks a truncTALE and the other two encode truncTALEs with short CRRs, no 28 aa repeat, and the highest level of sequence divergence from Tal2h in the N-terminal region; and Xoo MAFF 311018 harbors a disruptive IS element. These strains were incompatible, eliciting the HR. Since *Xo1*-mediated resistance is triggered by *X. oryzae* TAL effectors broadly and non-specifically (Triplett et al., 2016), the results of these inoculations reveal that resistance is being suppressed by each of the compatible strains, ostensibly by their respective truncTALEs, despite the diverse predicted DNA binding specificities represented. Suppression is nonetheless specific, because the NLR-type *R* gene *Rxo1* is effective against all the Asian Xoc strains, and the bacterial blight executor *R* genes *Xa7, Xa10*, and *Xa27*, and the pattern recognition receptor-like *R* gene *Xa21* are effective against one, the other, or both of the two Xoo strains tested that were compatible with Carolina Gold Select (PXO86 and PXO99A; Niño-Liu et al., 2006). As a direct additional test of specificity for Tal2h, we transformed the *Xa7*- and *Xa10*-incompatible Xoo strain PXO86 with the *tal2h* plasmid and inoculated the transformant to the near-isogenic rice varieties IRBB7 and IRBB10, which respectively carry those *R* genes, and to their parent IR24, which does not. There was no change in compatibility on IR24, and normal HR ensued on IRBB7 and IRBB10, confirming that Tal2h, and by extension each of the truncTALEs in the Carolina Gold Select-compatible strains, suppresses *Xo1*-mediated resistance specifically (Figure 2B).

### Impact of TruncTALE structural features on DNA binding

**Figure 3:**
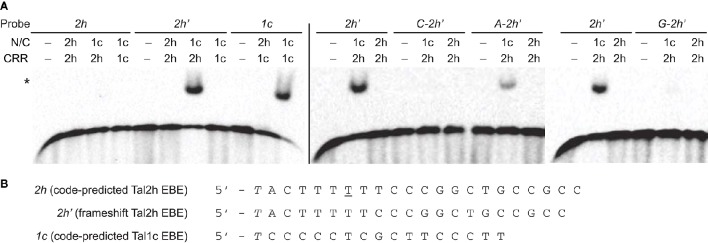
**Impact of structural features of Tal2h on DNA binding. (A)** Clones encoding the N-and C-terminal regions (N/C) of Tal2h (2h) and of the standard TAL effector Tal1c (1c), and clones encoding just the respective CRRs were constructed and combined to reconstitute the effectors or to create chimeras, and those proteins were tested for the ability to bind different double stranded DNA probes by electrophoretic mobility shift assay. At left, Tal2h, Tal1c, and a chimera with the N- and C-terminal regions of Tal1c and the CRR of Tal2h were tested for the ability to bind a probe containing the code-predicted EBE for Tal2h (*2h*) or a probe containing that sequence with a 1 bp deletion opposite the 28 aa repeat of Tal2h (the frameshift EBE; *2h'*). Also, Tal1c and a chimera of Tal2h with the Tal1c CRR were tested against a probe containing the code-predicted Tal1c EBE (*1c*). EBEs are preceded by a 5′ T. At right, Tal2h and the chimera composed of the N- and C-terminal regions of Tal1c with the CRR of Tal2h were tested against *2h'* with A, C, or G, substituted for the 5′ T, as indicated. Unmodified *2h'* was included as a control (repeated across gels). For all assays, probes (biotinylated double stranded DNA) were at 100 pM and were visualized by chemiluminescence using streptavidin-HRP conjugate. Proteins were at 300 nM. A no protein control (−) was included for each probe. The asterisk marks bound probe. **(B)** Nucleotide sequences of *2h, 2h'*, and *1c*.

The deletions in the N-terminal region of truncTALEs remove more than half of the first of the four cryptic repeats (repeat “−3”) and many of the positive residues in standard TAL effectors that might contribute to DNA binding (Supplementary Figure S2). Also, one of the conserved amino acid substitutions in truncTALEs is leucine in place of W232, a residue that contributes to the preference of TAL effectors for thymine immediately upstream of the CRR-encoded EBE. Furthermore, the 28 aa repeat type is distinct from the previously identified aberrant repeat types, which can disengage to accommodate a frameshift EBE (Richter et al., 2014). For these reasons, and because the apparent diverse DNA binding specificities of the CRRs suggested that specific DNA binding might not be important for truncTALE function, we questioned whether Tal2h could bind either a code-predicted EBE or a frameshift EBE. We tested this by electrophoretic mobility shift assay (EMSA). We also tested a chimera with the N- and C-terminal regions of the standard TAL effector Tal1c of BLS256 flanking the Tal2h CRR. We included assays of Tal1c and an inverse chimera (Tal2h containing the Tal1c CRR) against the Tal1c EBE. The results (Figure 3) revealed no binding by Tal2h or Tal2h with the Tal1c CRR to the corresponding EBEs, but binding by Tal1c with the Tal2h CRR to the frameshift Tal2h EBE. Notably, Tal1c with the Tal2h CRR did not bind the code-predicted Tal2h EBE without the frameshift. To account for the possibility that Tal2h prefers a base other than T preceding the EBE, binding of Tal2h to the frameshift EBE preceded by C, A, or G was also tested, with Tal1c containing the Tal2h CRR as a control. The chimera bound the frameshift EBE preceded by T as expected and bound weakly to that EBE preceded by A, but no binding by Tal2h was observed. DNA sequences were also assayed that tested whether Tal2h could bind (1) the code-predicted EBE preceded by a base other than T, (2) the EBE with a 1 bp insertion opposite the 28 aa repeat, (3) the EBE with A substituted for G at position 1, opposite the low specificity RVD NS, or (4) the EBE with an A to T substitution at position 7, which breaks up the potentially disruptive (Streubel et al., 2012) homopolymeric stretch of Ts from position 3 to position 8. Tal2h bound to none (Supplementary Figure S3 and Supplementary Table S4). Thus, the Tal2h CRR is operational, with the 28 aa repeat either obligately disengaging or forcing one of its neighbors to do so, but overall, the deletions and/or other polymorphisms at the N- or C- terminal regions (or at both) disallow DNA binding.

### Structural features of TruncTALEs important for suppression of *Xo1*-mediated resistance

**Figure 4:**
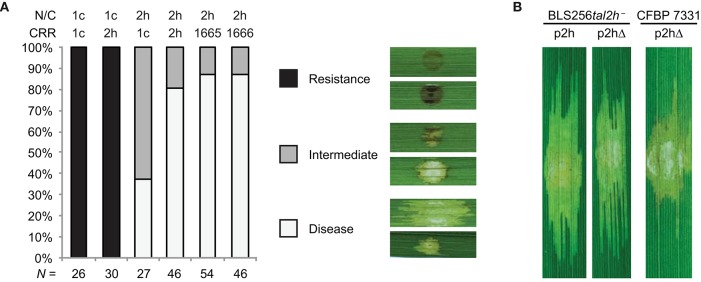
**Structural requirements for TruncTALE activity. (A)** Activity is influenced by the CRR but not by the presence of a 28 aa repeat. Constructs encoding Tal2h, standard TAL effector Tal1c, or CRR-swap chimeras, labeled as in Figure 3, were introduced into CFBP 7331 and assayed for their ability to suppress *Xo1*-mediated resistance by inoculation to Carolina Gold Select. Also tested were constructs that encode Tal2h containing the CRR of a Tal2h-based dTALE engineered to contain the 28 aa repeat, or one with a standard 34 aa repeat at that position instead (1665 and 1666, respectively; Supplementary Table S3). Multiple inoculations were done for each construct, and the result of each was scored as disease, resistance, or intermediate. The histogram shows the collated results. The total number (*N*) of inoculations scored for each construct is shown at bottom. Representative images are shown in the key at right. Images of all inoculated leaves are provided in Supplementary Figure S4. **(B)** The candidate NLS sequence of Tal2h is dispensable. Leaves of cultivar Carolina Gold Select were inoculated as in Figure 2 with Xoc BLS256 *tal2h* mutant M12 (BLS256*tal2h*^−^) or Xoc CFBP 7331 wild type, each transformed with a plasmid expressing Tal2h (p2h) or a plasmid expressing Tal2h missing the last seven amino acids, which include the candidate NLS (p2hΔ).

Next, we tested the functional importance of the CRR, the N- and C-terminal regions, and the 28 aa repeat type of truncTALEs by assaying chimeras of Tal2h, Tal1c, and Tal2h-based dTALEs with or without a 28 aa repeat. We also tested whether the candidate NLS sequence is required by assaying a Tal2h variant missing the last seven amino acids, which span it. Constructs were introduced into CFBP 7331 or, for the candidate NLS experiment, into the BLS256 *tal2h* mutant and CFBP 7331, and those transformants inoculated to Carolina Gold Select leaves (Figure 4A and Supplementary Figure S4, and Figure 4B, respectively). Leaves were scored for the development of watersoaked lesions that indicate compatibility and for the dark HR-like necrosis characteristic of *Xo1*-mediated resistance. In replicated assays, based on this scoring, reconstituted Tal2h suppressed *Xo1*-mediated resistance fully ~80% of the time, and partially ~20% of the time, while control Tal1c consistently did not suppress resistance. Tal1c harboring the Tal2h CRR also did not suppress resistance. The inverse chimera however, yielded an intermediate phenotype, completely suppressing resistance in roughly 40% of inoculations and suppressing it partially in the remaining ~60%. Tal2h carrying the CRR of a dTALE designed to match the encoded specificity of the Tal2h CRR, but assembled using only the four most common RVDs and with all repeats 34 aa in length, or of a matching dTALE engineered such that the sixth repeat is 28 aa, each suppressed resistance equally well and similarly to Tal2h. Similarly, Tal2h missing the candidate NLS sequence was unchanged in its ability to suppress resistance relative to wildtype Tal2h. Thus, features in the N- or C-terminal regions, or both, are essential for function, and the CRR can moderately influence suppressor activity, but the 28 amino acid repeat type and the candidate NLS sequence are dispensable.

## Discussion

Characterization of the ability of AvrBs4 of *X. euvesicatoria* to trigger resistance mediated by the Bs4 NLR protein in tomato first revealed a mode of action for TAL effectors distinct from direct gene activation (Schornack et al., 2004). The recently described elicitation of *Xo1*-mediated resistance by *X. oryzae* TAL effectors independent of an AD or any specific RVD sequence provides another example (Triplett et al., 2016). The simplest model for both of these is direct interaction between effector and R protein leading to activation of the R protein (Qi and Innes, 2013). Here we have described a new, contrasting example in which a member of a structurally distinct class of TAL effectors, truncTALEs, and ostensibly others in that class, specifically suppress resistance mediated by *Xo1*.

### Speculative model for suppression of *Xo1*-mediated resistance by a TruncTALE

Because truncTALEs lack an AD and, based on Tal2h, do not bind DNA or require their sole candidate NLS for activity, we surmise that a truncTALE suppresses resistance by means other than direct modification of host gene expression. Because suppression is specific to *Xo1*, we further surmise that the target of the suppressor activity is the immune receptor itself and not a downstream signaling component. *Xo1* has not been cloned, but based on its functional resemblance to *Bs4* and the abundance of NLR protein genes at the locus, it seems likely to encode an NLR protein that acts as the receptor or as part of a receptor complex. The observation that many distinct *X. oryzae* TAL effectors trigger *Xo1*-mediated resistance and that this elicitation does not depend on an AD or more than 3.5 repeats in the CRR supports a direct effector-receptor interaction model involving the conserved N-terminal region and conserved features of the repeat backbone (non-RVD) sequences early in the CRR (Triplett et al., 2016). Our CRR swap experiments between Tal2h and the standard TAL effector Tal1c showed that structures outside the CRR are essential for suppression. We speculate that Tal2h, and other truncTALEs that suppress *Xo1*-mediated resistance, do so by interacting directly with the receptor similarly to TAL effectors, and that the structural differences in the N-terminus lock the receptor in an inactive confirmation. The swap experiments also revealed however, that the CRR influences activity. Given the variation observed across ostensibly active truncTALEs (Figure 1C) this is unlikely to relate to overall RVD sequence but may depend on the sequence of RVDs, or the presence of particular RVDs, early in the CRR. Consistent with this notion, the RVD sequences of the non-active African truncTALEs diverge from those of Tal2h and the other, ostensibly active, Asian truncTALEs early, at position 3 (Figure 1). It is also plausible though that subtle differences in the repeat backbone sequences of active truncTALEs (and the dTALEs used) relative to standard TAL effectors, possibly early in the CRR, are responsible (Supplementary Figure S5). Consistent with this idea, African truncTALEs exhibit unique repeat backbone polymorphisms (Supplementary Figure S5). Also, repeat backbone sequences of TAL effectors that trigger *Xo1*-mediated resistance have some aa differences from those that do not (Triplett et al., 2016). We have not yet tested whether African truncTALEs trigger *Xo1*-mediated resistance. Their inability to suppress it may reflect an inability to interact in any way with the receptor, or instability. Notably, the African truncTALEs have shorter CRRs (six repeats) than the Asian truncTALEs, and have more amino acid substitutions in the N-terminal regions relative to Tal2h than any other truncTALE (Figure 1 and Supplementary Figure S1).

**Figure 5:**
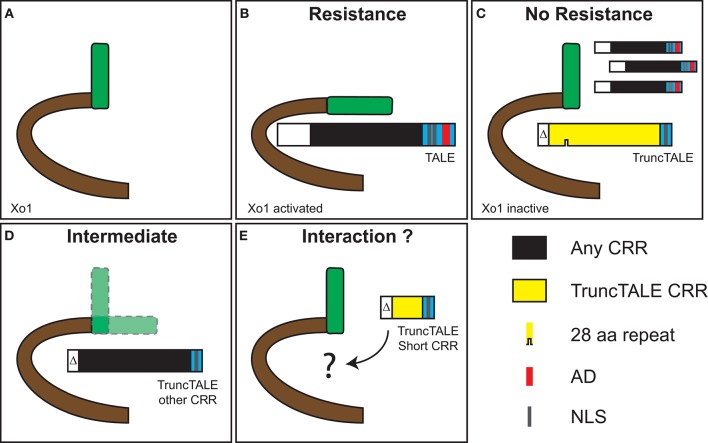
**Speculative model for suppression of *Xo1*-mediated resistance by a truncTALE. (A)** *Xo1* likely encodes an NLR protein (Triplett et al., 2016). **(B)** Recognition of *X. oryzae* TAL effectors by Xo1 activates the protein and leads to resistance. This recognition is independent of the TAL effector RVD sequence since several TAL effectors from *X. oryzae* pv. oryzicola and *X. oryzae* pv. oryzae trigger *Xo1*-mediated resistance (Triplett et al., 2016). We postulate that recognition takes place through direct interaction involving the TAL effector N-terminus and the beginning of the CRR, because TAL effectors with no AD and as few as 3.5 repeats trigger the resistance (Triplett et al., 2016). **(C)** A truncTALE can suppress *Xo1*-mediated resistance. Structural features outside the CRR, likely in the N-terminus, are essential for suppressor activity. We speculate that suppression also is through direct interaction, which maintains Xo1 in an inactive conformation. An active TruncTALE displaces TAL effectors as a result of greater affinity or possibly higher local titer, or both. **(D)** Substitution of the truncTALE CRR with a dissimilar one results in an intermediate phenotype, indicating compromised suppression. This suggests that features of the CRR influence suppressor activity. Given the variation observed across ostensibly active truncTALEs (Figure 1C) this is unlikely to relate to overall RVD sequence but may depend on the sequence of RVDs, or the presence of particular RVDs, early in the CRR. Alternatively, or additionally, truncTALE-specific polymorphisms in the backbone residues of the repeats, possibly those early in the CRR, may come into play (Supplementary Figure S5). Notably, the 28 aa repeat is not required for suppressor activity. **(E)** TruncTALEs with short CRRs, found in two African Xoc strains, lack suppressor activity. This strengthens the hypothesis that the RVDs early in the CRR influence activity, since these short CRR truncTALEs diverge from the others beginning at position 3 (Figure 1C). However, they also have more amino acid substitutions in the N-terminal regions relative to Tal2h than any other truncTALE (Figure 1C), and distinct repeat backbone polymorphisms, which might account for their lack of activity. It is also possible that these shorter proteins simply are not stably expressed.

Figure 5 provides a graphical representation of our speculative model for activation of *Xo1*-mediated resistance by TAL effectors and suppression of that resistance by a truncTALE. One might reasonably question whether a truncTALE somehow acts within the bacterial cell to prevent the triggering of *Xo1*-mediated resistance, rather than suppressing the resistance from within the plant cell. However, the presence of a truncTALE clearly does not interfere with delivery of TAL effectors that trigger *Xo1*-mediated resistance, e.g., AvrXa7 and AvrXa10 (Triplett et al., 2016; Figure 2B), and the N-terminal deletions in truncTALEs do not disrupt the T3S signal, arguing strongly against a role inside the bacterial cell. Indeed, co-infiltration of African Xoc strain CFBP 7331 carrying *tal2h* on a plasmid vector with CFBP 7331 carrying the empty vector resulted in watersoaking, not HR, on Carolina Gold Select (Supplementary Figure S6), confirming conclusively that Tal2h functions outside the bacterial cell.

### Lack of DNA binding

Our observation that Tal2h does not bind any of 11 tested probes in the EMSA but that its CRR swapped into a standard TAL effector is functional is an important underpinning of the protein-protein interaction model just described. The reciprocal CRR swap confirmed that structures outside of the CRR unique to truncTALEs ablate DNA binding. How truncations at the N- and C- terminal ends affect TAL effector DNA binding and activity has been intensively studied (Miller et al., 2011; Mussolino et al., 2011; Zhang et al., 2011; Meckler et al., 2013; Schreiber et al., 2015). Collectively, these studies have shown that, although required for activity, most of the C-terminal region is dispensable for DNA binding. Therefore, we would not expect the deletions in the C-terminal regions of truncTALEs to perturb the ability to bind DNA. In contrast, DNA binding has been shown to be sensitive to truncations at the N-terminus. For biotechnology, researchers have sought to delineate the minimal DNA binding domain of TAL effectors. Miller et al. (2011) found that the first 152 aa of TAL effectors could be removed, leaving 102 aa before the CRR, without negatively impacting activity, and this truncation has been widely used in custom TAL effector applications. Removal of an additional eight aa, however, reduces DNA binding affinity ~60-fold (Meckler et al., 2013). Thus, residues beginning somewhere between 152 and 160 are crucial for binding. The alignment between Tal2h and Tal1c shows that the second deletion in the N-terminal region of truncTALEs removes residues 159–173 (Supplementary Figure S1). This eliminates a large portion of cryptic repeat −3 including a stretch of positively charged residues on the inner side of the superhelix structure posited to be crucial for DNA binding (Supplementary Figure S2). This N-terminal deletion of truncTALEs, therefore, likely explains the inability to bind DNA. As noted earlier, a leucine substitution at W232 and possibly other substitutions may also negatively impact DNA binding.

### The 28 aa repeat

The CRR swap experiment that showed the Tal2h CRR to be functional also revealed that the 28 aa repeat either obligately disengages or forces one of its neighbors to do so: the chimera with the Tal2h CRR bound only a frameshift EBE missing the base opposite the 28 aa repeat. Because this base is in the middle of a stretch of six thymines, and the repeat on either side has a thymine-specific RVD, we were unable to determine from this experiment which repeat actually disengages. The six aa deletion at the second helix of the 28 aa repeat eliminates a hydrophobic patch at the interface with the next repeat that mediates inter-repeat stabilization, so it seems likely that either the 28 aa repeat or that neighbor, rather than the preceding neighbor, is the one to disengage. The obligate nature of the disengagement has not been observed before. Other aberrant repeat types characterized are able to bind the code-predicted EBE or a frameshift one (Richter et al., 2014). Conceivably, as a function of the neighboring RVDs, a 28 aa repeat at a different location in the CRR might be able to engage or disengage. The RVD itself might also have an impact. Further experimentation and ultimately structural elucidation will be necessary to fully characterize the behavior of this new repeat type. We found no 28 aa repeat in any available TAL effector sequence other than truncTALEs. However, the chimeric nature of Tal11g of Xoc strain CFBP 7342 and the presence of apparently chimeric truncTALE variants in Xoc strain BXOR1 and Xoo strains PXO86 and PXO99A, discussed earlier, underscore the possibility that a standard TAL effector bearing a truncTALE-like 28 aa repeat might be encountered. Understanding the behavior of this repeat, in different contexts and with different RVDs, will be important for identifying targets of any such effector.

### TruncTALEs and host-pathogen co-evolution

The ability conferred by *Xo1* (or an ancestral equivalent) to mount a defense response to TAL effectors as a class, independent of their individual DNA binding specificities, can be expected to have exerted strong selective pressure on *X. oryzae* to evolve a countermeasure, and thereby given rise to truncTALEs. Specific suppression of *Xo1*-mediated resistance by a truncTALE resembles its activation by a TAL effector in being apparently independent of the ability to bind a specific EBE. Almost certainly, the divergence of RVD sequences across truncTALEs, in light of our EMSA results, reflects the lack of any selection on these proteins to bind a specific DNA target in order to suppress resistance. In some populations, such as in Africa, or for some lineages, such as the one that gave rise to Japanese Xoo strain MAFF 311018, loss of selection for suppressor activity itself, or counterselection, might have resulted in divergence or disruption of the truncTALE gene sequences and consequent loss of activity. Clearly though, annotation of intact truncTALE coding sequences as pseudogenes should be discontinued and existing annotation corrected to reflect the fact that these coding sequences are functional.

The African Xoc strains and exceptional Asian Xoo strain notwithstanding, it is tempting to speculate that broad distribution of active truncTALEs in *X. oryzae* accounts for the relative paucity of NLR-type *R* genes identified to date that are effective against bacterial leaf streak or bacterial blight of rice. For comparison, of the 23 characterized genes for resistance to rice blast, caused by the fungus *Magnaporthe oryzae*, 21 encode NLR proteins (Liu et al., 2014). The rice genome (cv. Nipponbare) encodes as many as 480 NLR protein genes (Zhou et al., 2004). Because TAL effectors are by far the most abundant class of type III effector in *X. oryzae*, and are often important virulence factors, one might anticipate that NLR proteins capable of TAL effector recognition might be relatively abundant. Perhaps active truncTALEs broadly render such proteins ineffective. It will be of interest to determine whether *Xa1*-mediated resistance is defeated by truncTALEs. Notably, the *Xa1* gene is effective against Japanese race 1 strains such as MAFF 311018, in which the truncTALE gene promoter is disrupted by an IS element, but not against strains such as PXO86 and PXO99A, which each have an intact truncTALE gene.

### Future directions

We have presented a limited analysis of truncTALE diversity and function based on available sequences and strains, and a relatively low-resolution structure-function analysis. Our results nonetheless strikingly expand the paradigm for TAL effector mediated effects on the host, and raise a number of intriguing questions. These include what truncTALE representation is across a larger group of *X. oryzae* strains, whether truncTALEs are found outside *X. oryzae*, and what the detailed structural requirements for suppressor activity are. Perhaps most importantly, do active truncTALEs indeed interact directly with the immune receptor or receptor complex responsible for *Xo1*-mediated resistance, and if so, what is the molecular identity of that receptor, and how does the interaction differ from that with a standard TAL effector? Other areas of interest include whether the 28 aa repeat exists in any standard TAL effector, the structural basis for its requiring a frameshift EBE, and whether its behavior depends on its RVD and/or context. Yet another is the subcellular localization of truncTALEs. Is their candidate NLS functional? If not, is exclusion from the nucleus important for activity? And, can a truncTALE suppress Bs4-mediated resistance? The answers to these and related questions about this new class of TAL effectors and the scope of their activity will provide fundamental insight into the host pathogen interaction as well as practical information that will inform the development and strategic deployment of existing or engineered resistance genes. Approaches informed by a detailed understanding of truncTALE distribution and function promise better control not only of bacterial leaf streak and bacterial blight of rice, but potentially other economically important plant diseases caused by pathogens that depend on TAL effectors.

## Author contributions

AR, FR, LT, and AB conceived and designed the study. AR, FR, MH, and YH carried out the experiments. AR, FR, MH, LT, and AB analyzed and interpreted the data. AR, FR, MH, and AB prepared the paper, with assistance from LT and YH.

### Conflict of interest statement

The authors declare that the research was conducted in the absence of any commercial or financial relationships that could be construed as a potential conflict of interest.
