# Supplementary material for: Suppression of Xo1-Mediated Disease Resistance in Rice by a Truncated, Non-DNA-Binding TAL Effector of Xanthomonas oryzae
Source: Front Plant Sci. 2016 Oct 13;7:1516. doi: 10.3389/fpls.2016.01516 (PMC5062187; doi:10.3389/fpls.2016.01516)
Supplement: Supplementary file 1 [file Presentation1.pdf]

Supplementary Material

Suppression of *Xo1*-mediated disease resistance in rice by a truncated, non-DNA-binding TAL effector of *Xanthomonas oryzae*

Andrew C. Read[1], Fabio C. Rinaldi[1], Mathilde Hutin[1], Yong-Qiang He[1,2], Lindsay R. Triplett[3], and Adam J. Bogdanove[1]*

[1]Plant Pathology and Plant-Microbe Biology Section, School of Integrative Plant Science, Cornell University, Ithaca, NY, USA

[2]State Key Laboratory for Conservation and Utilization of Subtropical Agro-bioresources, The Key Laboratory of Ministry of Education for Microbial and Plant Genetic Engineering, and College of Life Science and Technology, Guangxi University, Nanning, China

[3]Department of Plant Pathology and Ecology, The Connecticut Agricultural Experiment Station, New Haven, CT 06511 USA

*** Correspondence:** Adam J. Bogdanove, Plant Pathology and Plant-Microbe Biology Section, School of Integrative Plant Science, Cornell University, 334 Plant Science Building, Ithaca, NY, 14853, USA.

ajb7@cornell.edu

A

```
BLS256_tal1c       1  MDPIRPRRPSPARELLPGPQPDRVQPTADRGVSAPAGSPLDGLPARRTVSRTRLPSPPAP
BLS256_tal2h       1  MDPIRSRTPSPAREPLPGPQPDRVQPTADRGVSAPAGGPLDALPARRTVSRTRLPSPPAP
BXOR1_tal11h       1  MDPIRSRTQSPAREGLPGPQPDRVQPTADRGVSAPAGSPLDGLPARRTVSRTRLPSPPAP
CFBP7331_tal8f     1  MDPIRPRRPSPAREPLPGPQPDRVQPTADRGVSAPAGSPLDGLPARRTVSRTRLPSPPAP
PXO86_tal6         1  MDPIRSRTPSPAREPLPGPQPDRVQPTADRGVSAPAGSPLDGLPARRTVSRTRLPSPPAP

BLS256_tal1c      61  SPAFSAGSFSDLLRPFDPSLLDTSLLDSMPAVGTPHTAAAPAEWDEAQSALRAADDPPPT
BLS256_tal2h      61  LPAFSAGSSTDRLRPFDPSLPDTSLFDSMPAVGTPHTEAAPA------------------
BXOR1_tal11h      61  LPAFSAGSSTDRLRPFDPSLPDTSLFDSMPAVGTPHTEAAPA------------------
CFBP7331_tal8f    61  LPAFSAGSFSDLLRPFDPSLPDTSLFDSMPAVGTPHTEAAPA------------------
PXO86_tal6        61  LPAFSAGSSTDRLRPFDPSLPDTSLFDSMPAVGTPHTEAAPA------------------

                                                          1        2
BLS256_tal1c     121  VRVAVTAARPPRAKPAPRRRAAQPSDASPAAQVDLRTLGYSQQQQEKIKPKVRSTVAQHH
BLS256_tal2h     103  -------------------------DTSPAAQVDLLTL---------------ATVAQHH
BXOR1_tal11h     103  -------------------------DTSPAAQVDLLTL---------------ATVAQHH
CFBP7331_tal8f   103  -------------------------DTSPAAQVDLLTL---------------ATVAQHH
PXO86_tal6       103  -------------------------DTSPAAQVDLLTL---------------ATVAQHH

                                                                         3
BLS256_tal1c     181  EALVGHGFTHAHIVALSQHPAALGTVAVTYQHIITALPEATHEDIVGVGKQWSGARALEA
BLS256_tal2h     123  EALVGHGFTHAHIVALSQHPAALGTVAVMYQHIITALPEATHEDIVGVGKQLSDARALEA
BXOR1_tal11h     123  EALVGHGFTHAHIVALSQHPAALGTVAVMYQDIITALPEATHEDIVGVGKQLSGARALEA
CFBP7331_tal8f   123  EALVGHGFTHAHIVALSQHPAALGTVAVTYQDIITALPEATHEDIVGVGKQLSGARALEA
PXO86_tal6       123  EALVGHGFTHAHIVALSQHPAALGTVAVTYQDIITALPEATHEDIVGVGKQLSGARALEA

BLS256_tal1c     241  LLTDAGELRGPPLQLDTGQLVKIAKRGGVTAMEAVHASRNALTGAPLN
BLS256_tal2h     183  LLTKAGELRGPPLQLDTGQLLKIARRGGVTAVEAVHAWRNALTGAPLN
BXOR1_tal11h     183  LLTKAGELRGPPLQLNTGQLLKIARRGGVTAVEAVHAWRNALTGAPLN
CFBP7331_tal8f   183  LLTKAGELRGPPLQLDTGQLLKIARRGGVTAVEAVHAWRNALTGAPLN
PXO86_tal6       183  LLTKAGELRGPPLQLDTGQLLKIARRGGVTAVEAVHAWRNALTGAPLN
```

B

```
BLS256_tal1c     1  IVAQLSRRDPALAALTNDHLVALACLGGRPALDAVKKGLPHAPEFIRRVNRRIAERTSHR
BLS256_tal2h     1  IVAQLSRRDPALAALTKDQLVALACFGGRPAPH---------------------------
BXOR1_tal11h     1  IVAQLSRRDPALAALTNDQLVALACLGGRPAPH---------------------------

BLS256_tal1c    61  VADYAHVVRVLEFFQCHSHPAHAFDEAMTQFGMSRHGLVQLFRRVGVTEFEARYGTLPPA
BLS256_tal2h    34  ------------------------------------------------------------
BXOR1_tal11h    34  ------------------------------------------------------------

BLS256_tal1c   121  SQRWDRILQASGMKRAKPSPTSAQTPDQTSLHAFADSLERDLDAPSPMHEGDQTRASSRK
BLS256_tal2h    34  ---------------------------------------------------------SRK
BXOR1_tal11h    34  ---------------------------------------------------------SRK

                      4
BLS256_tal1c   181  RSRSDRAVTGPSAQQAVEVRVPEQRDALHLPLSWRVKRPRTRIWGGLPDPGTPMAADLAA
BLS256_tal2h    37  RKSHD-------------------------------------------------------
BXOR1_tal11h    37  RKSHD-------------------------------------------------------

BLS256_tal1c   241  SSTVMWEQDADPFAGAADDFPAFNEEELAWLMELLPQ
BLS256_tal2h    41  -------------------------------------
BXOR1_tal11h    41  -------------------------------------
```

Supplementary Figure S1. Annotated amino acid sequence alignment of truncTALEs with Tal1c of Xoc strain BLS256. (**A**) The sequences of the N-terminal region (up to the CRR) of representatives from each TruncTALE N-terminal sequence group are shown aligned to that of the Xoc strain BLS256 TAL effector Tal1c. The green bar corresponds to the residues of cryptic repeat -3. Numbers above the alignment mark other features as follows: 1, the site of the truncation characterized by Miller and colleagues that is the basis for a TAL effector architecture widely used in biotechnology (Miller et al., 2011); 2, the site of the truncation characterized by Meckler and colleagues that resulted in an approximately 60-fold decrease in DNA binding affinity (Meckler et al., 2013); 3, the conserved tryptophan (W232) of standard TAL effectors that confers the preference for thymine immediately preceding the RVD-specified bases of the EBE (Schreiber and Bonas, 2014) and is substituted with leucine in truncTALEs. **(B)** The sequences of the C-terminal region of representatives from both TruncTALE C-terminal sequence groups are shown aligned to that of Tal1c. Red bars mark the residues of the two functional NLS of Tal1c and a blue bar marks the AD. A black bar marks the candidate NLS sequence present in the truncTALEs. The number 4 above the alignment marks the terminal position of the additional, truncTALE-like proteins encoded in PXO99A, PXO86, and BXOR1 (see text and Supplementary Table S6). Alignments were generated using T-coffee (Notredame et al., 2000) via the EMBL-EBI portal with default settings (Goujon et al., 2010) and visualized using BOXSHADE (http://www.ch.embnet.org/software/BOX_form.html). The allignment of the truncTALE terminal aspartic acid residue was adjusted by hand.

```
                               repeat -3
TALE        154 DLRTLGYSQQQQEKIKPKVRSTVAQHHEALVGHGF
truncTALE   111 DLLTL---------------ATVAQHHEALVGHGF

                       repeat -2
TALE        189 THAHIVALSQHPAALGTVAVKYQDMIAALPEA
truncTALE   131 THAHIVALSQHPAALGTVAVMYQHIITALPEA

                          repeat -1
TALE        221 THEAIVGVGKQWSGACALEALLTVAGELRGPPLQ
truncTALE   163 THEDIVGVGKQLSDARALEALLTKAGELRGPPLQ

                          repeat 0
TALE        255 LDTGQLLKIAKRGGVTAVEVHHTWRNALTGAPLN
truncTALE   197 LDTGQLLKIARRGGVTAVEAVHAWRNALTGAPLN
```

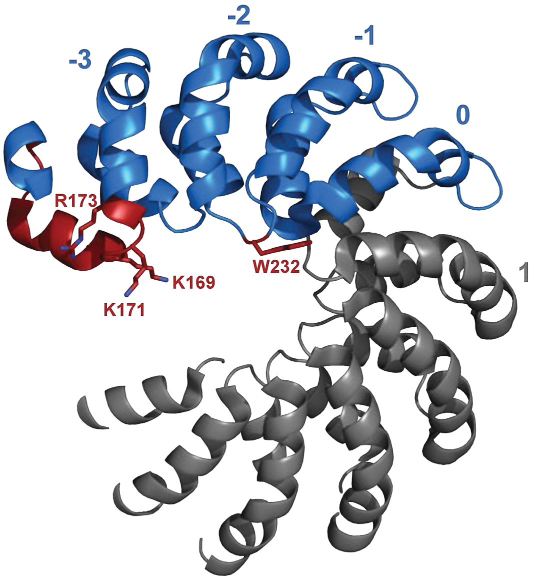

Supplementary Figure S2. Deletions in the N-terminal region of truncTALEs in relation to TAL effector structure. Alignment and structural representation highlighting the most significant differences (in red) between a standard TAL effector (TALE) and a truncTALE. The structure (PDB: 4HPZ) shows the cryptic repeats (-3 to 0, in blue) and the first several repeats of the CRR (in grey), with the residues that are missing in truncTALEs highlighted in red. This deletion disrupts cryptic repeat -3 and removes four positively charged residues at the DNA binding interface, labeled in the structure and underlined in the alignment. W232, responsible for the preference for thymine at position 0 of the EBE of standard TAL effectors and substituted with a leucine in truncTALEs is also labeled in the structure and is shown in red in the alignment. The structure is of dTALE2 which was assembled based on the sequence of AvrBs4 (Gao et al., 2012).

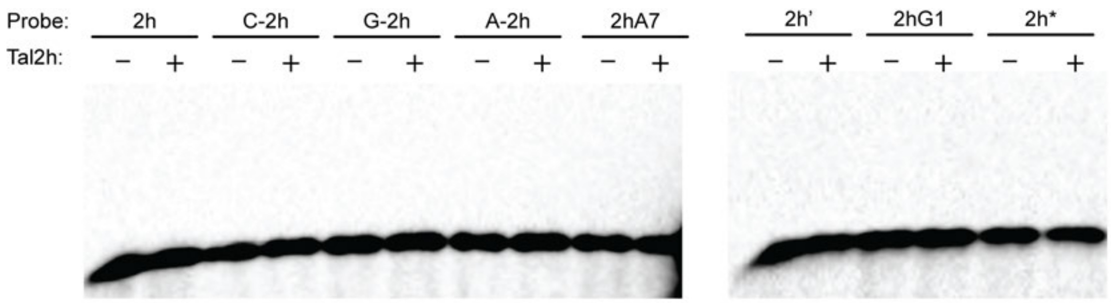

Supplementary Figure S3. EMSA for Tal2h interaction with additional EBE variants. Full-length Tal2h protein was assayed for the ability to bind any of eight different probes (Supplementary Table S4). For all assays, probes (biotinylated double stranded DNA) were at 100 pM and were visualized by chemiluminescence using streptavidin-HRP conjugate. Proteins were at 300 nM. A no protein control (–) was included for each probe. Tal2h shows no apparent binding to any of the probes.

Tal1c (Figure S4 part 1 of 6)

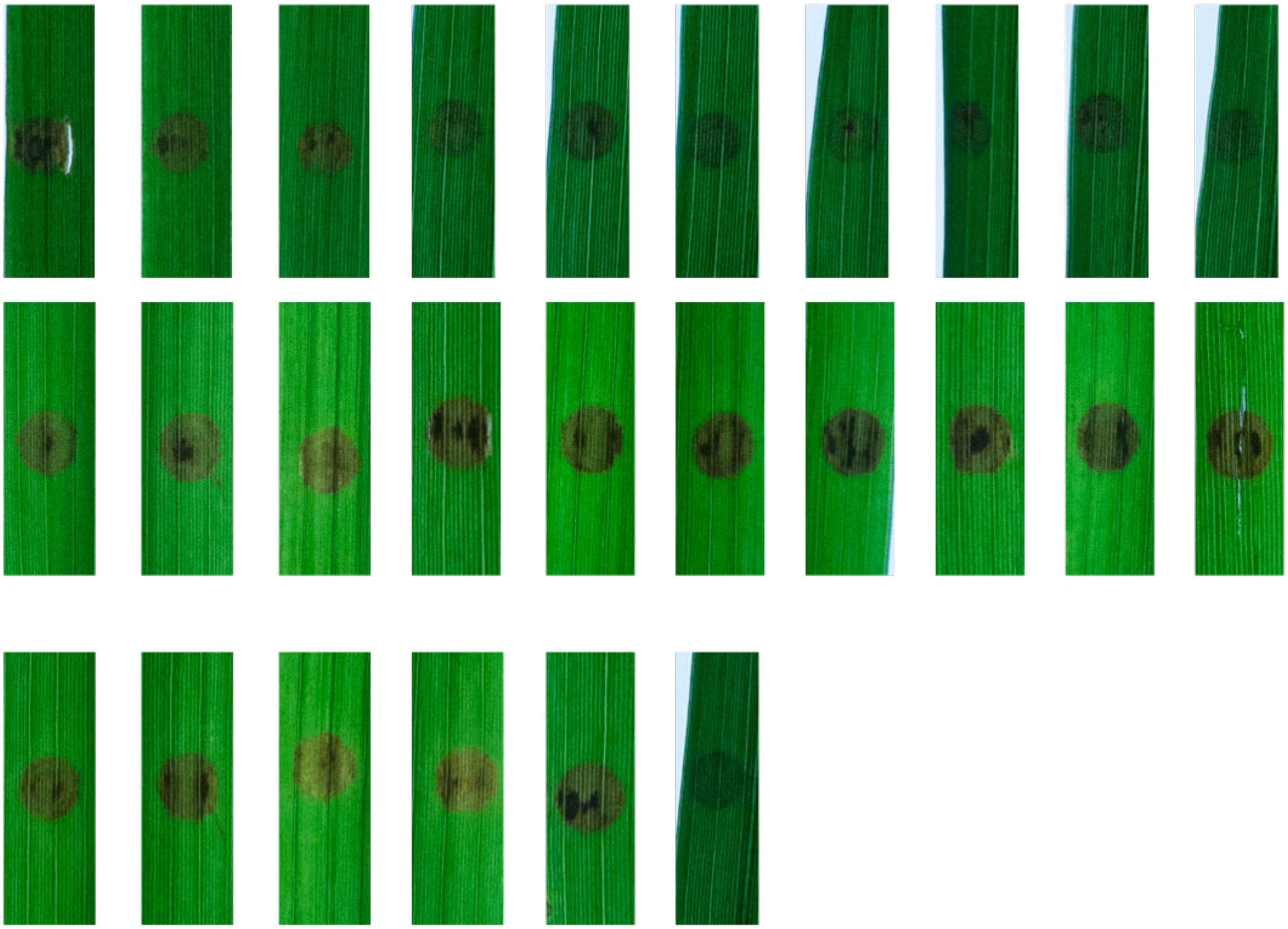

Tal1c N/C, Tal2h CRR (Figure S4 part 2 of 6)

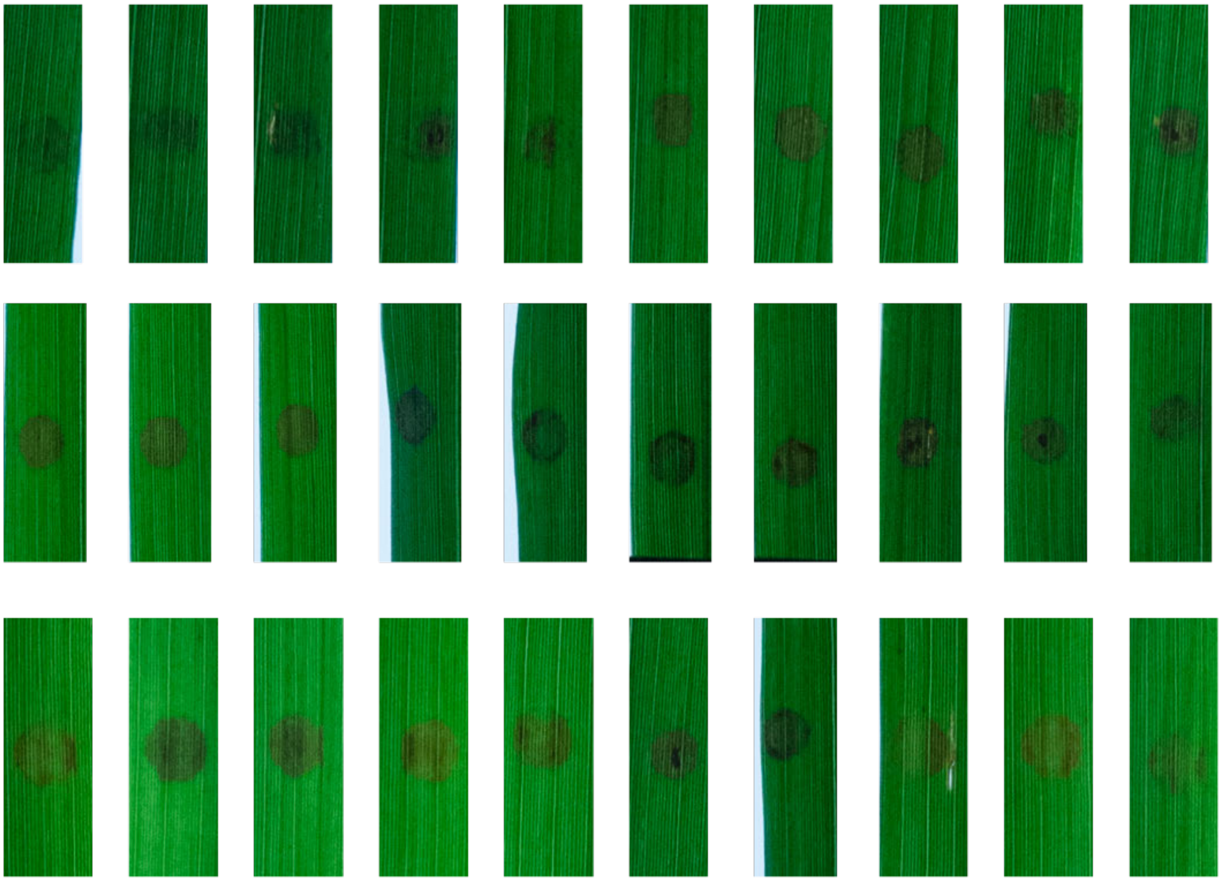

Tal2h N/C, Tal1c CRR (Figure S4 part 3 of 6)

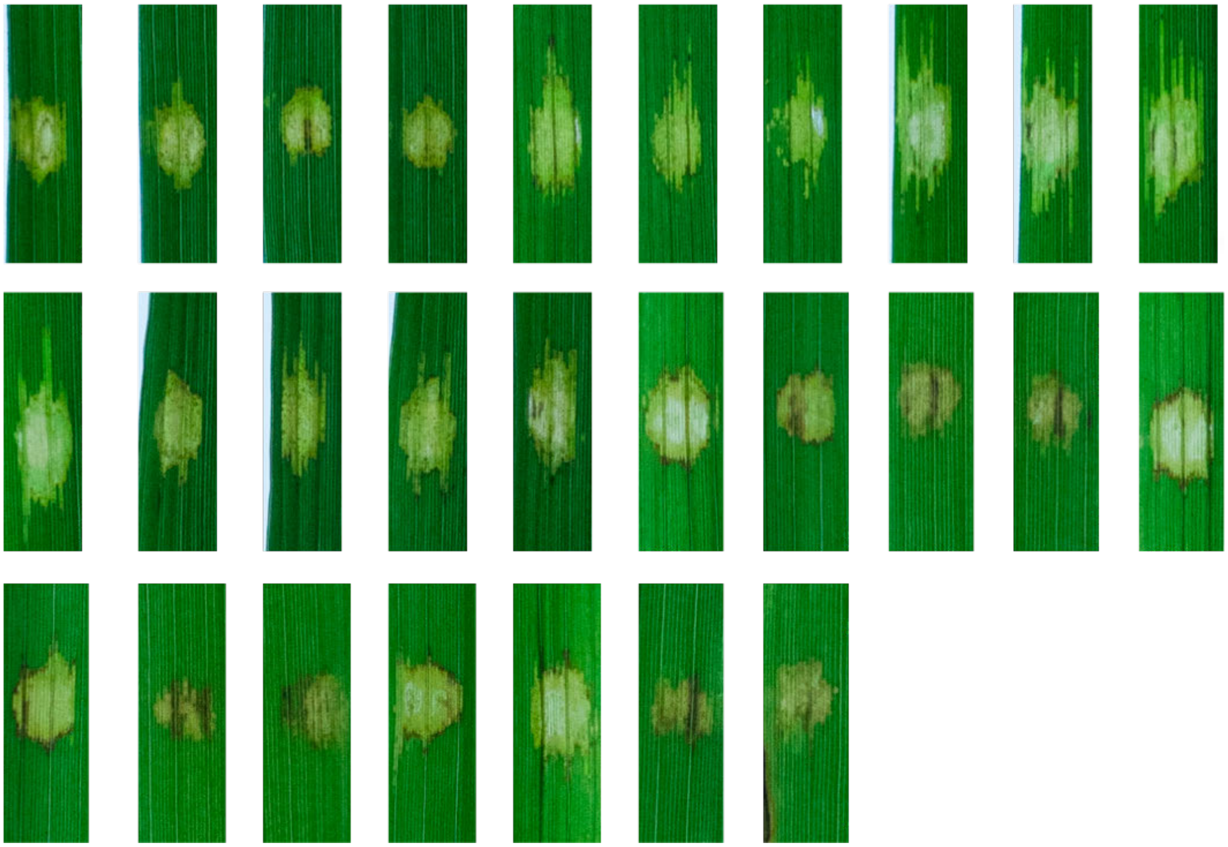

Tal2h (Figure S4 part 4 of 6)

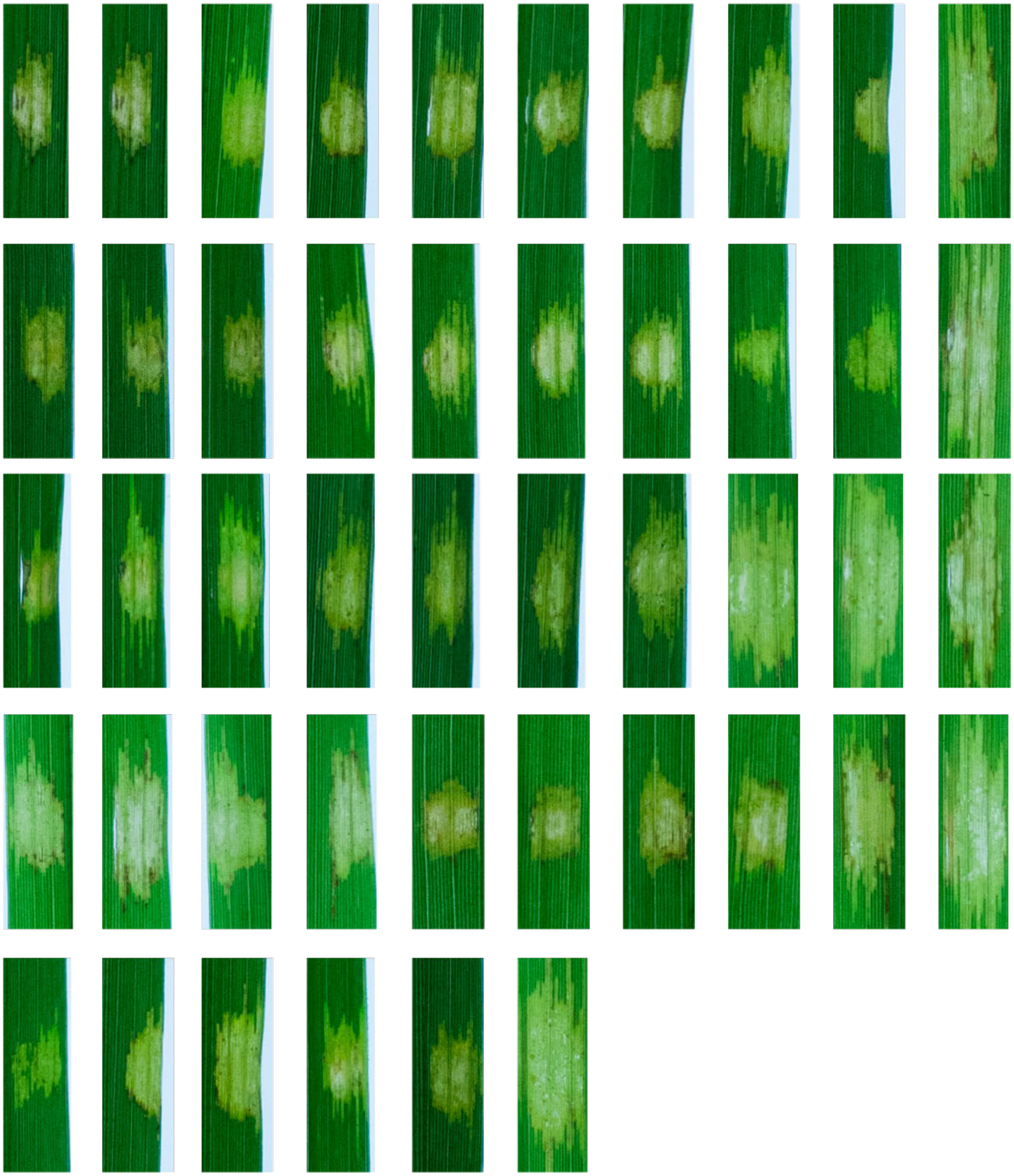

dT1665 (Figure S4 part 5 of 6)

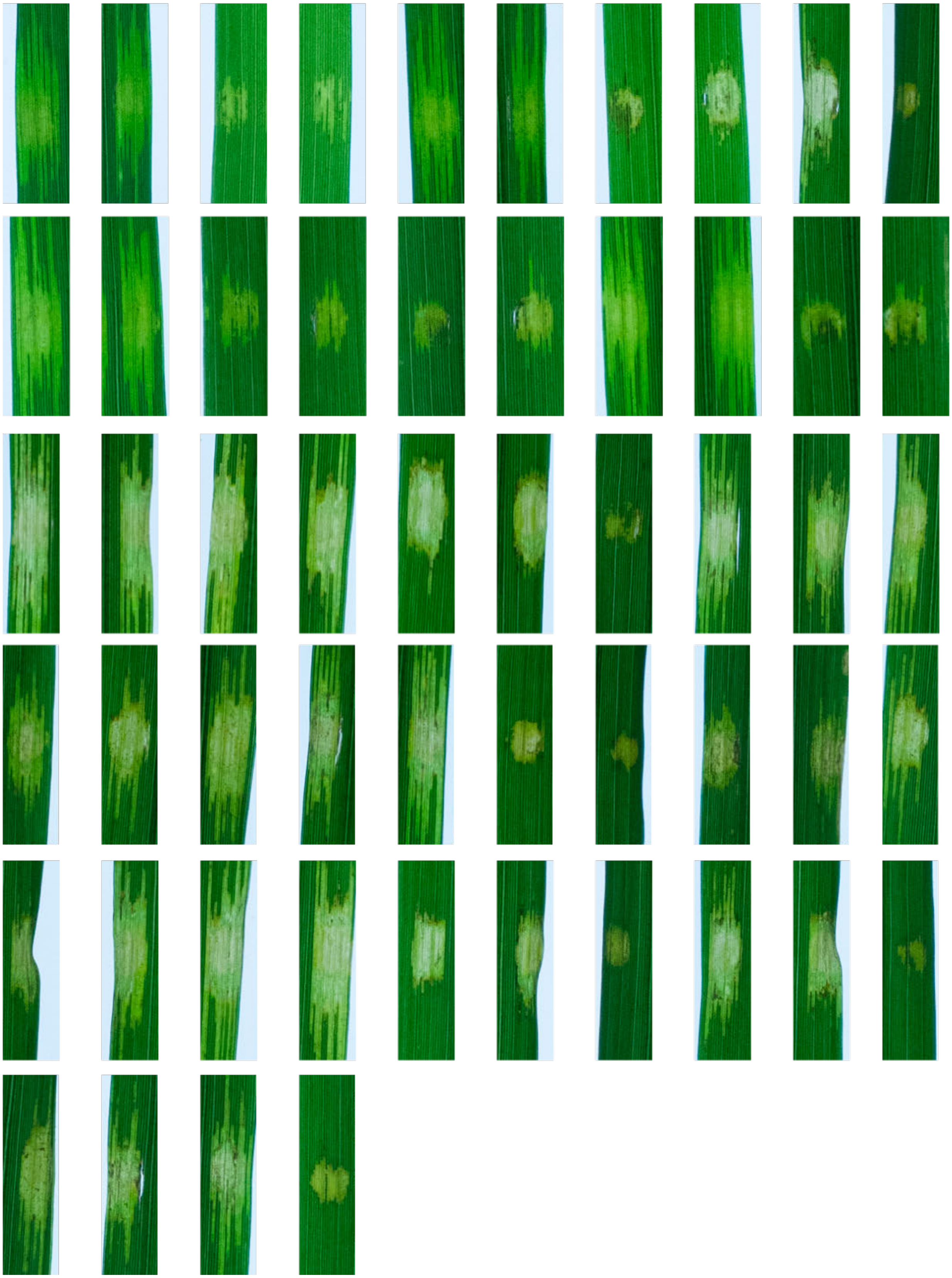

dT1666 (Figure S4 part 6 of 6)

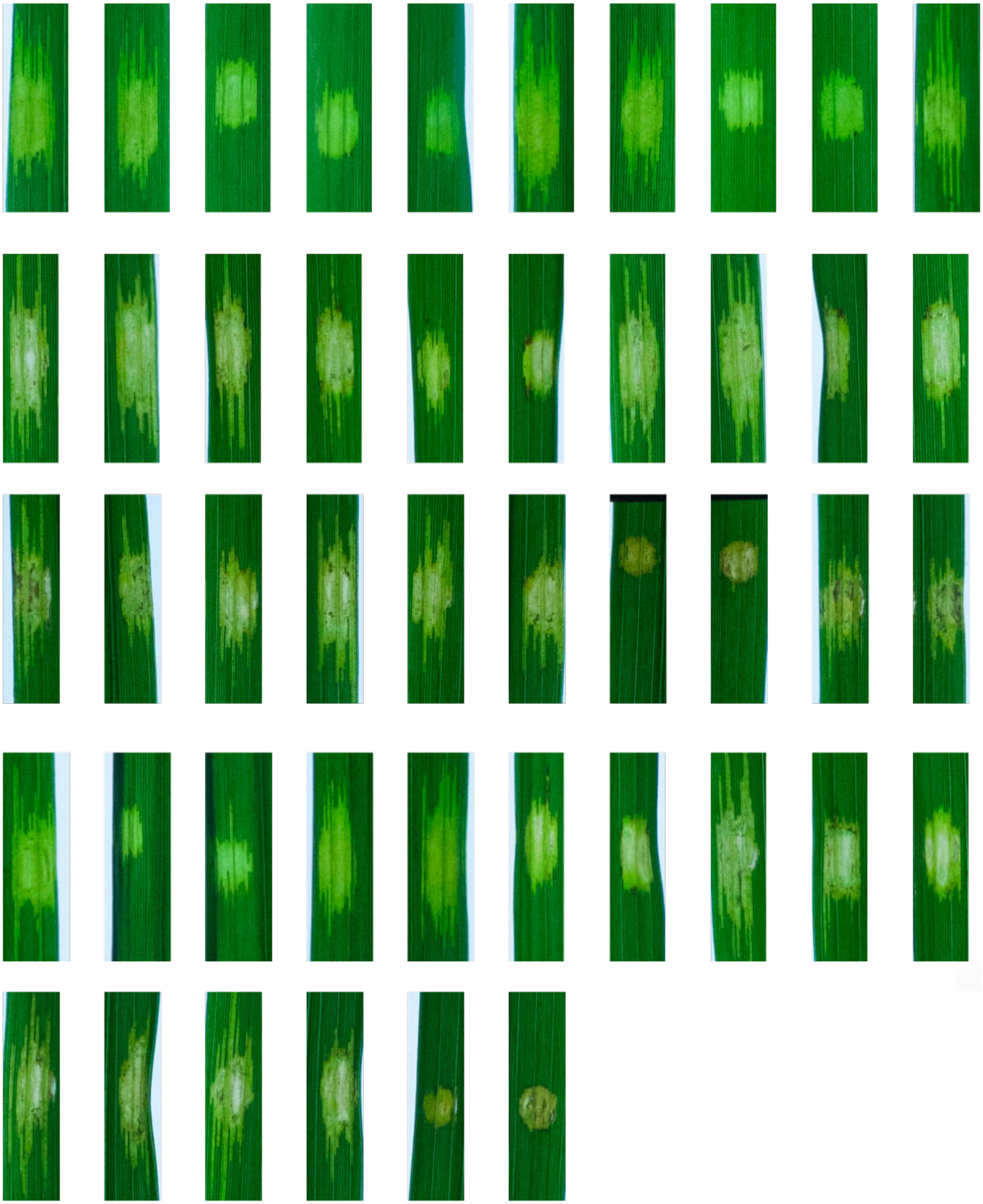

Supplementary Figure S4. All lesions scored for Figure 4A. Leaves were photographed on a light box at 8-10 days after infiltration.

BLS256 Tal1c CRR alignment

```
Tal1c_rpt1     1 LTPAQVVAIASHDGGKQALETVQRLLPVLCQDHG
Tal1c_rpt2     1 LTRDQVVAIASHDGGKQALETVQRLLPVLCQDHG
Tal1c_rpt3     1 LPPDQVVAIASHDGGKQALETVQRLLPVLCQDHG
Tal1c_rpt4     1 LTPDQVVAIASHDGGKQALETVQRLLPVLCQDHG
Tal1c_rpt5     1 LTPDQVVAIASHDGGKQALETVQRLLPVLCQDHG
Tal1c_rpt6     1 LTPDQVVAIASNGGGKQALETLQRLLPVLCQAHG
Tal1c_rpt7     1 LTPAQVVAIASHDGGKQALETVQRLLPVLCQAHG
Tal1c_rpt8     1 LTPDQVVAIANNNGGKQAVGTVQRLLPVLCQAHG
Tal1c_rpt9     1 LTPAQVVAIASHDGGKQALETVQRLLPVLCQAHG
Tal1c_rpt10    1 LTPDQVVAIASNGGGKQALETVQRLLPVLCQAHG
Tal1c_rpt11    1 LTLAQVVAIASHGGGKQALETVQRLLPVLCQAHG
Tal1c_rpt12    1 LTPDQVVAIANNNGGKQALETVQRLLPVLCQAHG
Tal1c_rpt13    1 LTPDQVVAIASHDGGKQALETVQRLLPVLCQAHG
Tal1c_rpt14    1 LTPAQVVAIASN-GGKQALETVRRLLPVLCQAHG
Tal1c_rpt15    1 LTPDQVVAIASNGGGKQALETVQRLLPVLCQDHG
```

BLS256 Tal2h CRR alignment

```
Tal2h_rpt1     1 LTPDQVVAIASNSGGKQALETVQRLLPVLCQDHG
Tal2h_rpt2     1 LTPDQVVAIASNDGGKQALETVQRLLPVLCQDHG
Tal2h_rpt3     1 LTPDQVVAIASHGGGKQALEAVQRLLPVLCQDHG
Tal2h_rpt4     1 LTPDQVVAIASHGGGKQALETVQRLLPVLCQDHG
Tal2h_rpt5     1 LTPDQVVAIASHGGGKQALEAVQRLLPVLCQAHG
Tal2h_rpt6     1 LTPDQVVAIASNGGGKQALETVLR------QAHG
Tal2h_rpt7     1 LTPDQVVAIASHGGGKQALETVQRVLPVLCQAHG
Tal2h_rpt8     1 LTLDQVAAIASHGGGKQALETVQRLLPVLCQAHG
Tal2h_rpt9     1 LTPDQVVAIASHDGGKQALETVQRLLPVLCQAHG
Tal2h_rpt10    1 LTPNQVVAIASHDGGKQALETVQRLLPVLCQAHG
Tal2h_rpt11    1 LTLDQVVAIASHDGGKQALETVQRLLPVLCQTHG
Tal2h_rpt12    1 LTPAQVVAIASNNGGKQALETVQRLLPMLCQAHG
Tal2h_rpt13    1 LTPAQVVAIASNNGGKQALETVQRLLPMLCQDHG
Tal2h_rpt14    1 LTPDQVVAIASHDSGKQALETMQRLLPVLCQDHG
Tal2h_rpt15    1 LTPDQVVAIASHGGGKQALETVQRLLPVLCQDHG
Tal2h_rpt16    1 LTPDQVVAIASHHGGKQALETVQRLLPVLCQDHG
Tal2h_rpt17    1 LTPDQVVAIASH-GGKQALETVQRLLPVLCQDHG
Tal2h_rpt18    1 LTPDQVVAIASH-GGKQALETVQRLLPVLCQDHG
Tal2h_rpt19    1 LTPDQVVAIASNNGGKQALETVRRLLQVLCQDHG
Tal2h_rpt20    1 LTPDQVVAIASHDGGKQALETVQRLLPVLCQAHG
```

CFBP 7331 Tal8f CRR alignment

```
Tal8f_rpt1     1 LTPDQVVAIASNIGGKQALETVQRLLPVLCQAHG
Tal8f_rpt2     1 LTPAQVVAIASHDGGKQALETVQRLLPVLCQDHG
Tal8f_rpt3     1 LTPDQVVAIASHDGGKQALETVQRLLPVLCQDHG
Tal8f_rpt4     1 LTPDQVVAIASN-GGKQALETVQRLLQVLCQDHD
Tal8f_rpt5     1 LTPDQVVAIASHDGGKQALETVQRLLPVLCQDHG
```

(Figure S5, continues on next page)

Golden Gate CRR alignment

```
GoldenGate_rpt1     1 LTPDQVVAIASNSGGKQALETVQRLLPVLCQDHG
GoldenGate_rpt2     1 LTPDQVVAIASHDGGKQALETVQRLLPVLCQDHG
GoldenGate_rpt3     1 LTPDQVVAIASNGGGKQALETVQRLLPVLCQDHG
GoldenGate_rpt4     1 LTPDQVVAIASNGGGKQALETVQRLLPVLCQDHG
GoldenGate_rpt5     1 LTPDQVVAIASNGGGKQALETVQRLLPVLCQDHG
GoldenGate_rpt6     1 LTPDQVVAIASNGGGKQALETVQRLLPVLCQDHG
GoldenGate_rpt7     1 LTPDQVVAIASNGGGKQALETVQRLLPVLCQDHG
GoldenGate_rpt8     1 LTPDQVVAIASNGGGKQALETVQRLLPVLCQDHG
GoldenGate_rpt9     1 LTPDQVVAIASHDGGKQALETVQRLLPVLCQDHG
GoldenGate_rpt1     1 LTPDQVVAIASHDGGKQALETVQRLLPVLCQDHG
GoldenGate_rpt1     1 LTPDQVVAIASHDGGKQALETVQRLLPVLCQDHG
GoldenGate_rpt1     1 LTPDQVVAIASNNGGKQALETVQRLLPVLCQDHG
GoldenGate_rpt1     1 LTPDQVVAIASNNGGKQALETVQRLLPVLCQDHG
GoldenGate_rpt1     1 LTPDQVVAIASHDGGKQALETVQRLLPVLCQDHG
GoldenGate_rpt1     1 LTPDQVVAIASNGGGKQALETVQRLLPVLCQDHG
GoldenGate_rpt1     1 LTPDQVVAIASNHGGKQALETVQRLLPVLCQDHG
```

Supplementary Figure S5. Alignment of the repeat sequences of Tal1c, an Asian truncTALE, an African truncTALE, and a dTALE. Repeat sequences of BLS256 Tal1c and Tal2h, CFBP 7331 Tal8f, and a representative dTALE (dT1665) built with our Golden Gate cloning kit (Cermak et al., 2011) were aligned (by protein, as labeled) using T-coffee (Notredame et al., 2000) via the EMBL-EBI portal with default settings (Goujon et al., 2010) and visualized using BOXSHADE (http://www.ch.embnet.org/software/BOX_form.html).

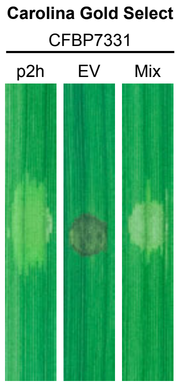

Supplementary Figure S6. Tal2h suppresses *Xo1*-mediated HR *in trans*. African Xoc strain CFBP 7331 carrying *tal2h* on a plasmid vector (p2h) and CFBP 7331 carrying the empty vector (EV) were infiltrated individually or together (Mix) into Carolina Gold Select leaves. Individual infiltrations were done at OD_{600} = 0.25. For the mix, suspensions at OD_{600} = 0.5 were mixed 1:1. Photographs were taken on a light box at 72 h after infiltration. Results for p2h and EV infiltrated individually at OD_{600} = 5 (not shown) were the same as those at OD_{600} = 0.25.

Supplementary Table S1. Strains and plasmids used.

Strain or plasmid	Origin or description[a]	Reference or source
X. oryzae pv. oryzicola		
B8-12	China	C. Song[b]
BLS256	Philippines	C.M. Vera Cruz[c]
BLS279	Philippines	C.M. Vera Cruz[c]
BXOR1	India	(Yashitola et al., 2000)
CFBP2286	Malaysia	CIRM-CFBP[d]
CFBP 7331 (MAI10)	Mali	(Gonzalez et al., 2007)
CFBP 7341 (BAI5)	Burkina Faso	(Wonni et al., 2011)
CFBP 7342 (BAI11)	Burkina Faso	(Wonni et al., 2014)
L8	China	(Zeng et al., 1995); Z. Yin[e]
M12	BLS256 marker exchange mutant with single disruptive insertion mapping to *tal2h*, Km[r]	(Cernadas et al., 2014); this study
RS105	China	(Fu et al., 1997); C. Song[b]
X. oryzae pv. oryzae		
MAFF311018	Japan race 1	(Ochiai et al., 2005)
PXO86	Philippines race 2	(Vera Cruz et al., 1984)
PXO99[A]	Philippines race 6	(Hopkins et al., 1992)
Plasmids		
pKEB31	pDD62 derivative containing Gateway destination vector cassette (Invitrogen) between *Xba*I and *Bam*HI sites, Tc[r]	(Cermak et al., 2011)
pAC99	pKEB31 containing *tal1c* of BLS256 missing the *Sph*I repeat-encoding fragment, Tc[r]	(Verdier et al., 2012)
pYH2	pKEB31 containing the *tal2h* BamHI fragment from EFC72, Tc[r]	This study
pKEB31-8-1c	pKEB31 containing Gateway fragment of pAR008-1c, Tc[r]	This study
pKEB31-8-2h	pKEB31 containing Gateway fragment of pAR008-2h, Tc[r]	This study
pKEB31-9-1c	pKEB31 containing Gateway fragment of pAR009-1c, Tc[r]	This study
pKEB31-9-2h	pKEB31 containing Gateway fragment of pAR009-2h, Tc[r]	This study
pKEB31-12-2h	pKEB31 containing Gateway fragment of pAR012-2h, Tc[r]	This study
pKEB31-8-dT1665	pKEB31 containing Gateway fragment of pAR008-dT1665, Tc[r]	This study
pKEB31-8-dT1666	pKEB31 containing Gateway fragment of pAR008-dT1666, Tc[r]	This study
pKEB31-9-dT1665	pKEB31 containing Gateway fragment of pAR009-dT1665, Tc[r]	This study
pHis_parallel-SNAP	Expression vector carrying a His tag upstream and a SNAP tag downstream of a multiple cloning site. Derivative of pHis_parallel1 (Sheffield et al., 1999). Expression driven by T7 promoter, Ap[r]	J. Lis[f]
pFR300	pHis_parallel_SNAP based vector containing coding sequence for the Tal2h N and C terminal regions without the CRR (fragment obtained from pAR008), Ap[r]	This study
pFR303	Version of pFR300 containing the coding sequence for the Tal2h CRR from pAR009-2h, Ap[r]	This study

pFR318	pHis_parallel_SNAP based vector containing coding sequence for truncated N- and C-termini (101 and 62 amino-acid residues from the N- and C-termini, respectively) of Tal1c, without the CRR, amplified from pTAL1 (Cermak et al., 2011), Ap^r	This study
pFR319	Version of pFR318 containing coding sequence for the Tal2h CRR from pAR009-2h, Ap^r	This study
pFR320	Version of pFR300 containing coding sequence for the Tal1c CRR from pAR009-1c, Ap^r	This study
pFR321	Version of pFR318 containing the coding sequence for the Tal1c CRR from pAR009-1c, Ap^r	This study
pAR008	Version of pTAL1 in which the Tal1c N and C terminal coding sequences have been replaced with those of Tal2h, gateway entry vector, Ap^r	This study
pAR009	Version of pTAL1 in which an *Sph*I site in the *tal1c* 3' end has been eliminated via silent mutation, gateway entry vector, Ap^r	This study
pAR012	Version of pAR008 carrying an introduced stop codon immediately before the coding sequence of the candidate NLS of Tal2h ('RKRKSHD'), gateway entry vector, Ap^r	This study
pNG7_28AA	Golden-gate module NG7 (Cermak et al., 2011) modified to create a Tal2h-like 28 amino acid repeat when combined with any module 6, Tc^r	This study
pCS468	Expression vector containing *tal1c* of BLS256, Sp^r	This study
EFC72	Cosmid containing a 39 kb fragment overlapping the BLS256 *tal2* cluster	This study
pAR008-1c	pAR008 containing the CRR encoding *Aat*II-*Sph*I fragment of *tal1c*, Ap^r	This study
pAR008-2h	pAR008 containing the CRR encoding *Aat*II-*Sph*I fragment of *tal2h*, Ap^r	This study
pAR009-1c	pAR009 containing the CRR encoding *Aat*II-*Sph*I fragment of *tal1c*, Ap^r	This study
pAR009-2h	pAR009 containing the CRR encoding *Aat*II-*Sph*I fragment of *tal2h*, Ap^r	This study
pAR012-2h	pAR012 containing the CRR encoding *Aat*II-*Sph*I fragment of *tal2h*, Ap^r	This study
pAR008-dT1665	pAR008 containing coding sequence of the Tal2h analog dT1665, with all standard repeats, Ap^r	This study
pAR008-dT1666	pAR008 containing coding sequence of the Tal2h analog dT1666, with a 28 aa repeat at position 6, Ap^r	This study
pAR009-dT1665	pAR009 containing coding sequence for dT1665, Ap^r	This study

[a] Ap^r, ampicillin resistance; Km^r, kanamycin resistance; Sp^r, spectinomycin resistance; Tc^r, tetracycline resistance

[b] Nanjing Agricultural University, Nanjing, China

[c] International Rice Research Institute, Los Baños, the Philippines

[d] International Centre for Microbial Resources – French Collection of Plant-associated Bacteria

[e] Temasek Laboratories, Singapore

[f] Cornell University

Supplementary Table S2. Primers used.

#	Sequence (5'-3')	Purpose
369	TTCTGCCCGGACCCCAACCGGATAG	Mapping M12
395	TCCCGTTGAATATGGCTCATAACACCCC	Mapping M12
397	GTCCACCTACAACAAAGCTCTCATCAACC	Mapping M12
1665	CTAGAGGTCTCACGGTGCTGCGGCAGGCGCATGGCCTGACCCCGGACCAAGTG GTGGCTATCGCCAGCAACGGTGGCGGCAAGCAAGCGCTCGAAACGAGACCC	Cloning, pNG7_28AA
1666	TCGAGGGTCTCGTTTCGAGCGCTTGCTTGCCGCCACCGTTGCTGGCGATAGCCA CCACTTGGTCCGGGGTCAGGCCATGCGCCTGCCGCAGCACCGTGAGACCT	Cloning, pNG7_28AA
1778	TGCAAAAGGGCGAATTCGGAGCCTG	Cloning, pAR008
1779	CCGCATTCAAGGAAGAGGAAATCGCATGATTGAAAGGGCGAATTCGACCCAGC TTTC	Cloning, pAR008
1781	TCCGAATTCGCCCTTTTGCATGTAAATAGGAGGTGCACCATGGATCCCA	Cloning, pAR008
1782	ATCATGCGATTTCCTCTTCCTTGAATGCGGGGCAGGACGTCCGCCGAGGCAG	Cloning, pAR008
1826	GCGATTTCCTCTTCCTTGAATGCGG	Mapping M12
2147	AAATAGGAGGTGCACCATGGATCCCATTCGTTCACGCAC	Cloning, pAR008
2148	AGCGGCGCCCGTCTCCAGGGGGGCACCCGTCAG	Cloning, pAR008
2149	GAGACGGGCGCCGCTACA	Cloning, pAR008
2150	GGTGCACCTCCTATTTACATGCAAAAGG	Cloning, pAR008
2168	TGAAAGGGCGAATTCGACCC	Cloning, pAR009
2169	TGAATGCGGGGCAGGACG	Cloning, pAR009
2171	AGGCGTCTTTACACGCATTCGCCGAT	Cloning, pAR012
2172	GATCCGGTGTTTGAGCTGAAG	Cloning, pAR012
2315	TAATAGGATCCAGTGGATCTACGCACGCTCGG	Cloning, pFR318
2316	TATATACTAGTTTCAAACGCGATGGGACGTGCG	Cloning, pFR318
2359	TATATATGGATCCCATTCGTTCACG	Cloning, pFR300
2360	TATATACTAGTTTCAATCATGCGATTTCCTCTTCCTTG	Cloning, pFR300

Supplementary Table S3. RVD sequences of Tal2h and Tal2h analogous dTALEs.

Protein	1	2	3	4	5	6[a]	7	8	9	10	11	12	13	14	15	16	17	18	19	20	21
Tal2h	NS	ND	HG	HG	HG	NG	HG	HG	HD	HD	HD	NN	NN	HD	HG	HH	H*	H*	NN	HD	H*
dT1665	NS	HD	NG	NG	NG	NG	NG	NG	HD	HD	HD	NN	NN	HD	NG	NH	HD				
dT1666	NS	HD	NG	NG	NG	NG	NG	NG	HD	HD	HD	NN	NN	HD	NG	NH	HD				

[a] Purple shaded boxes with white text represent 28 aa repeats.

Supplementary Table S4. EMSA probes used.

Probe	Sequence[a]
2h	5′ – cggcccgacgTACTTTTTTCCCGGCTGCCGCCatgtcatgtc – 3′
	3′ – gccgggctgcATGAAAAAAGGGCCGACGGCGGtacagtacag – 5′-Biotin
2h′	5′ – cggcccgacgTACTTT-TTCCCGGCTGCCGCCatagccggtc – 3′
	3′ – gccgggctgcATGAAA-AAGGGCCGACGGCGGtatcggccag – 5′-Biotin
1c	5′ – cggcccgacgTCCCCCTCGCTTCCCTTatgtcatgtc – 3′
	3′ – gccgggctgcAGGGGGAGCGAAGGGAAtatcggccag – 5′-Biotin
C-2h′	5′ – cggcccgacg**C**ACTTT-TTCCCGGCTGCCGCCatagccggtc – 3′
	3′ – gccgggctgcGTGAAA-AAGGGCCGACGGCGGtatcggccag – 5′-Biotin
A-2h′	5′ – cggcccgacg**A**ACTTT-TTCCCGGCTGCCGCCatagccggtc – 3′
	3′ – gccgggctgcTTGAAA-AAGGGCCGACGGCGGtatcggccag – 5′-Biotin
G-2h′	5′ – cggcccgacg**G**ACTTT-TTCCCGGCTGCCGCCatagccggtc – 3′
	3′ – gccgggctgcCTGAAA-AAGGGCCGACGGCGGtatcggccag – 5′-Biotin
C-2h	5′ – cggcccgacg**C**ACTTTTTTCCCGGCTGCCGCCatagccggtc – 3′
	3′ – gccgggctgcGTGAAAAAAGGGCCGACGGCGGtatcggccag – 5′-Biotin
G-2h	5′ – cggcccgacg**G**ACTTTTTTCCCGGCTGCCGCCatagccggtc – 3′
	3′ – gccgggctgcCTGAAAAAAGGGCCGACGGCGGtatcggccag – 5′-Biotin
A-2h	5′ – cggcccgacg**A**ACTTTTTTCCCGGCTGCCGCCatagccggtc – 3′
	3′ – gccgggctgcTTGAAAAAAGGGCCGACGGCGGtatcggccag – 5′-Biotin
2hA7	5′ – cggcccgacgTACTTTT**A**TCCCGGCTGCCGCCatagccggtc – 3′
	3′ – gccgggctgcATGAAAATAGGGCCGACGGCGGtatcggccag – 5′-Biotin
2hG1	5′ – cggcccgacgT**G**CTTTTTTCCCGGCTGCCGCCatgtcatgtc – 3′
	3′ – gccgggctgcACGAAAAAAGGGCCGACGGCGGtacagtacag – 5′-Biotin
2h*	5′ – cggcccgacgTACTTT**G**TTTCCCGGCTGCCGCCatgtcatgtc – 3′
	3′ – gccgggctgcATGAAACAAAGGGCCGACGGCGGtacagtacag – 5′-Biotin

[a] Nucleotide modifications at the binding sites relative to the code-predicted target (2h) are in bold. A dash indicates deletion of the nucleotide at that specific position relative to 2h.

Supplementary Table S5. Genome coordinates of coding sequences for truncTALEs and truncTALE-related sequences represented in Figure 1C and Figure 1D.

Strain	Gene	Genome GenBank ID	Coordinates
BLS256	*tal2h*	NC_017267.2	1528794-1531670
BLS279	*tal5g*	NZ_CP011956.1	1545821-1548713
CFBP 2286	*tal2j*	NZ_CP011962.1	1567344-1570236
L8	*tal5i*	NZ_CP011960.1	1547239-1550131
B8-12	*tal5i*	NZ_CP011955.1	1547863-1550959
RS105	*tal5e*	NZ_CP011961.1	1547866-1550962
BXOR1	*tal11h*	NZ_CP011957.1	3210814-3213403
CFBP 7331	*tal8f*	NZ_CP011958.1	3458708-3460091
CFBP 7341	*tal8f*	NZ_CP011959.1	3410078-3411461
MAFF 311018	ψ*tal5*[a]	NC_007705.1	2999333-3001924
PXO86	*tal6*	NZ_CP007166.1	2812524-2815116
PXO99A	*tal3b*	NC_010717.2	1864304-1866895
BXORI	*tal12*[b]	NZ_CP011957.1	3373285-3375499
CFBP 7342	ψ*tal11g*[c]	NZ_CP007221.1	3264795-3266637
PXO86	*tal3*[b]	NZ_CP007166.1	2020886-2023901
PXO99A	*tal3a*[b]	NC_010717.2	1859069-1862083

[a] Contains an insertion sequence element in the promoter region, likely rendering it a pseudogene (Ψ).
[b] Encodes a protein with a Tal2h-like N-terminus and a standard TAL effector C-terminus except for a few substitutions in the first NLS and a premature stop just after that.
[c] Contains an insertion sequence element in the 5' end of the coding sequence, rendering it a pseudogene (Ψ).

References

Cermak, T., Doyle, E.L., Christian, M., Wang, L., Zhang, Y., Schmidt, C., Baller, J.A., Somia, N.V., Bogdanove, A.J., and Voytas, D.F. (2011). Efficient design and assembly of custom TALEN and other TAL effector-based constructs for DNA targeting. *Nucleic Acids Res.* 39**,** e82.

Cernadas, R.A., Doyle, E.L., Nino-Liu, D.O., Wilkins, K.E., Bancroft, T., Wang, L., Schmidt, C.L., Caldo, R., Yang, B., White, F.F., Nettleton, D., Wise, R.P., and Bogdanove, A.J. (2014). Code-assisted discovery of TAL effector targets in bacterial leaf streak of rice reveals contrast with bacterial blight and a novel susceptibility gene. *PLoS Path.* 10**,** e1003972.

Fu, Z., Zhou, Y., Xu, Z., Yang, J., Fu, Z.Q., Zhou, Y.Z., Xu, Z.G., and Yang, J. (1997). A study on the relationship between resistance to two bacterial diseases in rice varieties. *Jiangsu J. Agric. Sci.* 13**,** 157-161.

Gao, H., Wu, X., Chai, J., and Han, Z. (2012). Crystal structure of a TALE protein reveals an extended N-terminal DNA binding region. *Cell Res.* 22**,** 1716-1720.

Gonzalez, C., Szurek, B., Manceau, C., Mathieu, T., Sere, Y., and Verdier, V. (2007). Molecular and pathotypic characterization of new *Xanthomonas oryzae* strains from West Africa. *Mol. Plant-Microbe Interact.* 20**,** 534-546.

Goujon, M., Mcwilliam, H., Li, W., Valentin, F., Squizzato, S., Paern, J., and Lopez, R. (2010). A new bioinformatics analysis tools framework at EMBL-EBI. *Nucleic Acids Res.* 38**,** W695-699.

Hopkins, C.M., White, F.F., Choi, S.H., Guo, A., and Leach, J.E. (1992). Identification of a family of avirulence genes from *Xanthomonas oryzae* pv. oryzae. *Mol. Plant-Microbe Interact.* 5**,** 451-459.

Meckler, J.F., Bhakta, M.S., Kim, M.S., Ovadia, R., Habrian, C.H., Zykovich, A., Yu, A., Lockwood, S.H., Morbitzer, R., Elsaesser, J., Lahaye, T., Segal, D.J., and Baldwin, E.P. (2013). Quantitative analysis of TALE-DNA interactions suggests polarity effects. *Nucleic Acids Res.* 41**,** 4118-4128.

Miller, J.C., Tan, S., Qiao, G., Barlow, K.A., Wang, J., Xia, D.F., Meng, X., Paschon, D.E., Leung, E., Hinkley, S.J., Dulay, G.P., Hua, K.L., Ankoudinova, I., Cost, G.J., Urnov, F.D., Zhang, H.S., Holmes, M.C., Zhang, L., Gregory, P.D., and Rebar, E.J. (2011). A TALE nuclease architecture for efficient genome editing. *Nat. Biotechnol.* 29**,** 143–148.

Notredame, C., Higgins, D.G., and Heringa, J. (2000). T-Coffee: a novel method for fast and accurate multiple sequence alignment. *J. Mol. Biol.* 302**,** 205-217.

Ochiai, H., Inoue, Y., Takeya, M., Sasaki, A., and Kaku, H. (2005). Genome sequence of *Xanthomonas oryzae* pv. oryzae suggests contribution of large numbers of effector genes and insertion sequences to its race diversity. *Japan Agric. Res. Q.* 39**,** 275-287.

Schreiber, T., and Bonas, U. (2014). Repeat 1 of TAL effectors affects target specificity for the base at position zero. *Nucleic Acids Res.* 42**,** 7160-7169.

Sheffield, P., Garrard, S., and Derewenda, Z. (1999). Overcoming expression and purification problems of RhoGDI using a family of "parallel" expression vectors. *Protein Expression Purif.* 15**,** 34-39.

Vera Cruz, C.M., Gossele, F., Kersters, K., Segers, P., Van Den Mooter, M., Swings, J., and De Ley, J. (1984). Differentiation between *Xanthomonas campestris* pv. oryzae, *Xanthomonas campestris* pv. oryzicola and the bacterial 'brown blotch' pathogen on rice by numerical analysis of phenotypic features and protein gel electrophoregrams. *J. Gen. Microbiol.* 130**,** 2983-2999.

Verdier, V., Triplett, L.R., Hummel, A.W., Corral, R., Cernadas, R.A., Schmidt, C.L., Bogdanove, A.J., and Leach, J.E. (2012). Transcription activator-like (TAL) effectors targeting *OsSWEET* genes enhance virulence on diverse rice (*Oryza sativa*) varieties when expressed individually in a TAL effector-deficient strain of *Xanthomonas oryzae*. *New Phytol.* 196**,** 1197-1207.

Wonni, I., Cottyn, B., Detemmerman, L., Dao, S., Ouedraogo, L., Sarra, S., Tekete, C., Poussier, S., Corral, R., Triplett, L., Koita, O., Koebnik, R., Leach, J., Szurek, B., Maes, M., and Verdier, V. (2014). Analysis of *Xanthomonas oryzae* pv. oryzicola population in Mali and Burkina Faso reveals a high level of genetic and pathogenic diversity. *Phytopathology* 104**,** 520-531.

Wonni, I., Ouedraogo, L., and Verdier, V. (2011). First report of bacterial leaf streak caused by *Xanthomonas oryzae* pv. oryzicola on rice in Burkina Faso. *Plant Dis.* 95**,** 72-73.

Yashitola, J., Reddy, A.P.K., and Sonti, R.V. (2000). A widely distributed lineage of *Xanthomonas oryzae* pv. *oryzae* in India may have come from native wild rice. *Plant Dis.* 84**,** 465-469.

Zeng, X., Lai, W., and Xu, D. (1995). Serological specificity of leaf streak pathogen of rice. *J. South China Agric. Univ.* 16**,** 65-68.
